# K27-linked RORγt ubiquitination by Nedd4 potentiates Th17-mediated autoimmunity

**DOI:** 10.1186/s12929-025-01120-2

**Published:** 2025-02-19

**Authors:** Qiuming Zeng, Hui Guo, Na Tang, Pranav S. Renavikar, Nitin J. Karandikar, Amy E. Lovett-Racke, Michael K. Racke, Chengkai Yan, Rong Tang, Sushmita Sinha, Krishnendu Ghosh, Jeremy P. Ryal, Song Ouyang, Min Chen, Foued Amari, Coppola Vincenzo, R. Marshall Pope, Yalan Li, Huan Yang, Wallace Y. Langdon, Jian Zhang

**Affiliations:** 1 Department of Neurology, Xiangya Hospital, Central South University, Changsha, Hunan 410008 People’s Republic of China; 2 Department of Pathology, The University of Iowa Roy J. and Lucille A. Carver College of Medicine, Iowa City, IA 52242 USA; 3 Department of Microbial Infection and Immunity, The Ohio State University, Columbus, OH 43210 USA; 4 Department of Neurology, The Ohio State University, Columbus, OH 43210 USA; 5 Genetically Engineered Mouse Modeling Core, The Ohio State University, Columbus, OH 43210 USA; 6 Department of Cancer Biology and Genetics, The Ohio State University, Columbus, OH 43210 USA; 7 Proteomics Facility, The University of Iowa Roy J. and Lucille A. Carver College of Medicine, Iowa City, IA USA; 8 School of Biomedical Sciences, The University of Western Australia, Perth, Australia; 9 Clinical Research Center for Neuroimmune and Neuromuscular Disorders, Xiangya Hospital, Central South University, Changsha, Hunan 410008 People’s Republic of China; 10 National Clinical Research Center for Geriatric Disorders, Xiangya Hospital, Central South University, Changsha, Hunan 410008 People’s Republic of China

**Keywords:** Nedd4, HECT E3 ubiquitin ligase, T helper cell 17, RORγt, K27 polyubiquitination, Experimental autoimmune encephalitis

## Abstract

**Background:**

The HECT E3 ubiquitin ligase Nedd4 has been shown to positively regulate T cell responses, but its role in T helper (Th) cell differentiation and autoimmunity is unknown. Th17 cells are believed to play a pivotal role in the development and pathogenesis of autoimmune diseases. Nevertheless, the regulation of RORγt activation during Th17 cell differentiation by TCR signaling is yet to be elucidated. These uncharted aspects inspire us to explore the potential role of Nedd4 in Th17-mediated autoimmunity.

**Methods:**

We evaluated the impact of Nedd4 deficiency on mouse T cell development and differentiation using flow cytometry and siRNA transfection, and subsequently validated these findings in T cells from patients with multiple sclerosis (MS). Furthermore, we investigated the influence of Nedd4 deficiency on Th17-mediated autoimmunity through experimental autoimmune encephalomyelitis (EAE), a mouse model of MS. Subsequently, we elucidated the molecular mechanism underlying the interaction between Nedd4 and RORgt through immunoprecipitation, mass spectrometry analysis, and lentiviral transduction. Additionally, we identified Nedd4 as an E3 ubiquitin ligase for RORγt. Moreover, we characterized the tyrosine residue sites and polyubiquitination patterns involved in RORγt ubiquitination.

**Results:**

In this study, we report that loss of Nedd4 in T cells specifically impairs pathogenic and non-pathogenic Th17 responses, and Th17-mediated EAE development. At the molecular level, Nedd4 binds to the PPLY motif within the ligand binding domain of RORγt, and targets RORγt at K112 for K27-linked polyubiquitination, thus augmenting its activity.

**Conclusion:**

Nedd4 is a crucial E3 ubiquitin ligase for RORγt in the regulating Th17 cell development and offers potential therapeutic benefits for treating Th17-mediated autoimmune diseases.

**Supplementary Information:**

The online version contains supplementary material available at 10.1186/s12929-025-01120-2.

## Background

IL-17-producing CD4^+^ T helper (Th) cells are defined by specific developmental and functional features that are distinct from “classical” Th1 and Th2 cells [1, 2]. Th17 cells produce primarily IL-17A and IL-17F, which promote local chemokine production to recruit monocytes and neutrophils to the sites of inflammation. Studies of infectious diseases highlight the critical role of the Th17 response in host defense against fungal and bacterial pathogens [3]. However, by amplifying inflammation, Th17 cells are thought to play a key role in the development and pathogenesis of autoimmune diseases [4, 5], including multiple sclerosis (MS) and its murine model, experimental autoimmune encephalomyelitis (EAE) [6, 7]. In mice, Th17 cells develop from naïve CD4^+^ T cells in the presence of TGF-β and IL-6 (for non-pathogenic Th17) or IL-1β, IL-6, and IL-23 (for pathogenic Th17) [8–10], which induces the expression of RORγt, the master transcription factor that controls Th17 cell differentiation via a Stat3-dependent mechanism [11, 12]. RORγt is an orphan receptor, and has been considered as a critical regulator of anti-microbial immunity and a major target in the fight against inflammatory pathologies [13]. The regulation of RORγt activation during Th17 cell differentiation by TCR signaling is unknown.

Multiple post-translational modifications, as well as interactions with various co-factors, modulate RORγt function [12]. One of the post-translational modifications is ubiquitination, which is essential for regulating T cell function [14, 15]. It has been shown that several ubiquitin ligases including TRAF5 [16] and Itch [17] might target RORγt for K63- or K48-linked ubiquitination, thus regulating RORγt expression or function. Unlike the studies mentioned above, we will further employ the mice expressing inactive forms of E3 ligases and its primary T cells to better elucidate the ubiquitination process of RORγt.

Nedd4 (also known as Nedd4-1, neuronal precursor cell-expressed developmentally down-regulated 4) is a HECT-type E3 ubiquitin ligase which is comprised of a C-terminal HECT domain and an N-terminal C2 domain, and three (mouse) or four (human) WW domains. The C2 domain regulates cellular localization, the WW domains provide substrate recognition, typically by binding to the PY motif (L/PPxY), and the HECT domain confers E3 ligase activity and has a ubiquitin binding surface that enables progressivity of ubiquitination [18, 19]. Nedd4 has been shown to positively regulate T cell activation [20, 21], but the roles of Nedd4 in T helper (Th) cell differentiation and autoimmunity are unknown.

In this study, we found that although Nedd4 deficiency does not impair differentiation of Th1, Th2 and inducible regulatory T cells (iTregs), Th17 cell differentiation is greatly compromised in the absence of Nedd4 under both non-pathogenic and pathogenic Th17-polarizing conditions. In support of this observation, mice deficient for Nedd4, or deficient for Nedd4 in T cells, develop ameliorated EAE with an impaired antigen-specific Th17 response. In addition, we found that CD4^+^ T cells from patients with MS express heightened levels of NEDD4 and RORγt. At the molecular level, Nedd4 WW domains bind to the PPLYKEL motif, an extended Nedd4 WW domain-binding motif, at the carboxyl terminus of the RORγt ligand-binding domain (LBD), and targets RORγt for K27-linked polyubiquitination at K112 which enhances its activity. This ubiquitination is abrogated in T cells lacking Nedd4 or expressing Nedd4 C854A, an E3 dead mutant. Furthermore, targeting Nedd4 by in vitrol delivery of Nedd4 siRNA attenuates Th17 responses of MS patients. Therefore, our study identified a novel function for Nedd4 in Th17 cell differentiation, and Th17-mediated autoimmunity. Our data indicate that targeting Nedd4 may represent a novel therapeutic approach for EAE, and possibly for MS in humans.

## Materials and methods

### Mice

C57BL/6 mice (Stock No: 000664), *Rosa26-Cre-ER*^*T2*^ mice (Stock No: 008463), *Cd4 Cre* mice (Stock No: 017336), *LysM Cre* mice (Stock No: 004781), *Cd11c Cre* mice (Stock No: 008068), *Cd19 Cre* mice (Stock No: 004126), *Rorc Cre* mice (Stock No: 022791), *Tcrb*^–/–^ mice (Stock No: 002118), and *Rorc*^–/–^ (Stock No: 007571) were acquired from the Jackson laboratories. The mice bearing LoxP-flanked alleles encoding Nedd4 or Nedd4-2 (called ‘*Nedd4*^*f/f*^ mice’ and ‘*Nedd4-2*^*f/f*^ mice’ here, respectively) were generated in-house (see METHOD DETAILS). We generated knockin mice expressing RORγt Y479F (*Rorc*^*Y479F*^) and expressing Nedd4 C854A (equivalent to human Nedd4 C867A; *Nedd4*^*C854A*^) by CRISPR/Cas9 technology (see METHOD DETAILS). We crossed *Nedd4*^*f/f*^ mice with *Rosa26-Cre-ER*^*T2*^ mice, *Cd4 Cre*, *LysM Cre*, *Cd11c Cre*, and *Cd19 Cre* mice to generate *Nedd4*^*f/f*^*Rosa26-Cre-ER*^*T2*^ mice (called *'Nedd4*^*CreER*^* mice*' thereafter), *Cd4 Cre-Nedd4*^*f/f*^*, LysM Cre-Nedd4*^*f/f*^*, Cd11c Cre-Nedd4*^*f/f*^*,* and *Cd19 Cre-Nedd4*^*f/f*^ mice, respectively. *Nedd4*^*CreER*^ mice were i.p. injected with 2 mg of tamoxifen (T5648; Sigma-Aldrich, St. Louis, MO) or corn oil for 5 continuous days, and rested for 7 days before use. We also crossed *Nedd4*^*f/f*^ mice with *Rorc Cre* mice to generate *Rorc Cre*-*Nedd4*^*f/f*^ mice. *Cd4 Cre-Nedd4-2*^*f/f*^ mice were generated by crossing *Nedd4-2*^*f/f*^ mice with *Cd4 Cre* mice*.* For all mouse studies, > 3 mice were used for each experimental cohort per specified genotype. All mice were bred and housed under specific pathogen–free conditions. All mice were used for experiments at ages of 8 to 12 wk. All experimental protocols followed National Institutes of Health guidelines.

### Human specimens

MS patients (treatment-naïve and in remission) and healthy controls were recruited from the Ohio State University Wexner Medical Center and the University of Iowa Hospitals and Clinics. MS patients (treatment-naïve and in relapse) were recruited from Xiangya Hospital of Central South University. Peripheral blood mononuclear cells (PBMCs) were isolated by Ficoll density-gradient centrifugation and frozen in media containing DMSO for future use.

### Antibodies and reagents

Antibodies used for the western blot were: RORγt (clone AFKJS-9, catalog no. 14–6988-82, eBioscience); RORα (clone C-7, catalog no. sc-518081, Santa Cruz Biotechnology); RORγt (clone B2D, catalog no. 14–6981-82, eBioscience); Nedd4-2 (clone EPR8269(2), catalog no. ab168349, Abcam); Nedd4 (catalog no. 21698–1-AP, Proteintech); Nedd4 (clone H-135, catalog no. sc-25508, Santa Cruz Biotechnology); Itch (clone H-110, catalog no. sc-25625, Santa Cruz Biotechnology); Itch (clone G-11, catalog no. sc-28367, Santa Cruz Biotechnology); FLAG (clone M2, catalog no. F1804, Sigma-Aldrich); Myc(9E10, ab32, Abcam); HA (clone F-7, catalog no. sc-7392, Santa Cruz Biotechnology); Anti-mouse IgG, HRP-linked Antibody (catalog no. 7076, Cell Signaling Technology); Anti-rabbit IgG, HRP-linked Antibody (catalog no. 7074, Cell Signaling Technology); Monoclonal Anti-β-Actin antibody produced in mouse (catalog no. A2228, Sigma-Aldrich); Anti-Ubiquitin (linkage-specific K27) antibody (catalog no. ab181537, Abcam); Ub antibody (clone P4D1, catalog no. sc-8017, Santa Cruz Biotechnology). Antibodies used for flow cytometry were: CD8-APC/Cy7 (clone 53–6.7, catalog no. 100714, BioLegend); CD4-Pacific Blue (clone GK1.5, catalog no. 100428, BioLegend); TCR-β-PE/Cy7 (clone H57-597, catalog no. 109222, BioLegend); CD69-PE (clone H1.2F3, catalog no. 104508, BioLegend); CD44-PE (clone IM7, catalog no. 103024, BioLegend); CD25-FITC (clone PC61, catalog no. 102005, BioLegend); CD62L-FITC (clone MEL-14, catalog no. 104405, BioLegend); CD4-FITC (clone GK1.5, catalog no. 100406, BioLegend); IFN-γ-APC (clone XMG1.2, catalog no. 505810, BioLegend); IL-17-PE (clone TC11-18H10.1, catalog no. 506904, BioLegend); CD25-PE (clone PC61, catalog no. 102005, BioLegend); Foxp3-Pacific Blue (clone MF-14, catalog no. 126409, BioLegend); CD4-APC (clone GK1.5, catalog no. 100412, BioLegend); I-A^b^-MOG_35-55_-tetramer-PE (catalog no. TS-M704-1, Medical & Biology laboratories). Antibodies used in cell culture: CD3 (clone 145-2C11, catalog no. 830301, BioLegend); IFN-γ (clone R4-6A2, catalog no. 505702, BioLegend); CD28 (clone 37.51, catalog no. 14–0281-82, eBioscience); IL-4 (clone PA5-25,165, catalog no. PA5-25,165, Invitrogen). Critical commercial assays: Naïve CD4^+^ and CD4^+^ T Cell Isolation Kits (catalog no. 130–104-453, Miltenyi Biotec); Mouse Regulatory T Cell Staining Kit #1 (catalog no. 88–8111-40, eBioscience); Mouse IL-17A ELISA MAX™ Deluxe Sets (catalog no. 432504, BioLegend); Mouse IL-6 ELISA MAX™ Deluxe Sets (catalog no. 431304, BioLegend); Mouse GM-CSF ELISA MAX™ Deluxe Sets (catalog no. 432204, BioLegend); Mouse IL-10 ELISA MAX™ Deluxe Sets (catalog no. 431414, BioLegend); Mouse IFN-γ ELISA MAX™ Deluxe Sets (catalog no. 430804, BioLegend); LEGEND MAX™ Mouse IL-21 ELISA Kit (catalog no. 446107, BioLegend); LEGEND MAX™ Mouse IL-17F ELISA Kit (catalog no. 436107, BioLegend); Silver Stain for Mass Spectrometry kit (catalog no. 24600, Pierce Biotechnology). Corn oil, Tamoxifen, Complete Freund’s Adjuvant, Phorbol-12-myristate-13-acetate (PMA), Ionomycin, Percoll were obtained from Sigma-Aldrich. IL-12, TGF-β, mIL-6, mIL-23, IL-1β were obtained from Abcam. mIL-2, IL-4, Collagenase (Type II) powder were obtained from Gibco. Fixation/Permeabilization Concentrate, Permeabilization Buffer (10X), CFSE were obtained from eBioscience. Mycobacterium tuberculosis strain H37Ra was obtained from Difco. Myelin oligodendrocyte glycoprotein peptide 35–55 was obtained from GL Biochem. Pertussis toxin was obtained from List Biological Laboratories. DNase I was obtained from Roche. Cycloheximide (CHX) was obtained from Thermo Scientific. Protein A Sepharose CL-4B was obtained from GE Health Care. Polybrene was obtained from Santa Cruz Biotechnology.

### Generation of *Nedd4* and *Nedd4-2* conditional knockout strains, *Rorc*^*Y479F*^ and *Nedd4*^*C854A*^ knockin mice

The Nedd4 conditional knockout mice (cKO) were generated at Taconic Biosciences (Cranbury, NJ, USA) by targeting C57BL/6 embryonic stem (ES) cells and followed by blastocyst microinjection for subsequent chimera generation. The targeting vector was constructed for conditional deletion of exons 2 and 3 of mouse *Nedd4* gene in a plasmid containing two LoxP sequences upstream and downstream of the target region. The long homologous arm was isolated from intron 1 and the short homologous aim was isolated from intron 3. Deletion of exons 2 and 3 led to the removal of 239 bp coding sequence and generated a premature stop codon in the *Nedd4* mRNA, thus resulting in the loss of functional Nedd4 protein. Moreover, the *Nedd4* mRNA with such a deletion was degraded due to non-sense-mediated mRNA decay [22]. Targeted clones were confirmed by Southern blot for proper homologous recombination with single integration of the Neo-resistance cassette, which was flanked by two Frt sequences. The targeting vector was electroporated into C57BL/6 ES cells which were selected with G418 using standard protocols. Two confirmed ES cell clones (A7 and F1) were injected into the mouse BALB/c blastocysts. Breeding of the male chimeras of A7 line to Flp-deleter female mice (7089-F; Taconic), resulted in germline transmission combined with successful deletion of the Neo-resistance gene. Conditional KO Nedd4 (*Nedd4*^*f/f*^*)* mice were crossed with *Rosa26-Cre-ER*^*T2*^ mice (in which sequence encoding a fusion of Cre recombinase and the estrogen receptor (ER^T2^) was recombined into the ubiquitously expressed Rosa26 locus) to generate *Nedd4*^*f/f*^*Rosa26-Cre-ER*^*T2*^ mice (called *'Nedd4*^*CreER*^* mice*' here). *Nedd4*^*f/f*^ mice were also crossed with *Cd4 Cre, LysM Cre, Cd11c Cre* and *Cd19 Cre* mice to generate *Cd4 Cre-Nedd4*^*flf*^*, LysM Cre-Nedd4*^*f/f*^, *Cd11c Cre-Nedd4*^*f/f*^*,* and *Cd19 Cre-Nedd4*^*fl/f*^ mice to specifically delete Nedd4 in T cells, myeloid cells, DCs, and B cells, respectively.

C57BL/6N*-Nedd4l*^*tm1a(KOMP)Wtsi/H*^ mice were purchased from Knockout Mouse Project (KOMP) Repository at the University of California at Davis (Project ID: CSD4676) which were further crossed to *ACTBFLPe* (*ACTB:FLPe B6J*) mice to delete LacZ/Neo cassettes and obtain the clean tm1c allele according to the breeding strategies recommended by the International Mouse Phenotyping Consortium. The LoxP flanked *Nedd4-2* allele (*Nedd4-2*^*f/f*^) mice were crossed to *Cd4 Cre* mice to generate *Cd4 Cre-Nedd4-2*^*f/f*^ mice.

*Nedd4*^*C854A*^ and *Rorc*^*Y479F*^ mice were both generated by the Genetically Engineered Mouse Modeling Core of the Ohio State University Comprehensive Cancer Center using CRISPR/Cas9 technology. The online algorithm at www.benchling.org was used to design the CRISPR/Cas9 targeting strategy. GeneArt Platinum Cas9 Nuclease protein (B25642) was purchased from ThermoFisher Scientific (Waltham, MA, USA). The mix of assembled single guide RNA with Cas9 protein complexes (RNP) and ssODN was microinjected into C57Bl/6Tac zygotes. C57Bl/6Tac WT animals were used for founder mating purposes. Synthetic tracrRNA and crRNA were purchased from Sigma-Aldrich (St. Louis, MO, USA).

TracrRNA to target *Nedd4*^*C854A*^: GGGTAAAACAGCTACTCACC(AGG). TracrRNA(PAM) to target *Rorc*^*Y479F*^: CGCCTTCCCTCCACTCTATA(AGG). The two customized synthetic single strand oligo donor DNAs (ssODNs) bearing the desired mutations were purchased from Integrated DNA Technologies (IDT, Coralville, Iowa, USA).

ssODN to knockin the mutation *Nedd4*^*C854A*^: GCTCGAATGGACCACAATCCTTCACAGTGGAACAATGGGGCACCCCTGATAAGCTGCCAAGAGCACACACAGCGTGAGTAGCTGTTTTACCCTAGACAGGGCATAGTTTCTCCCTGCTGGTGTAGGGGCAGCCCCAGCTGTGCCCTCTTG.

ssODN to knockin the mutation *Rorc*^*Y479F*^:

AAAGGAAAACTCCGGAGCCTGTGCAGCCAACATGTGGAAAAGCTGCAGATCTTCCAGCACCTCCACCCCATCGTGGTCCAAGCCGCCTTCCCTCCACTCTTTAAAGAACTCTTCAGCACTGATGTTGAATCCCCTGAGGGGCTGTCAAAG

The presence of the desired mutation was confirmed by Sanger sequencing. *Nedd4*^*C854A*^ mice are genotyped by PCR amplifying a 223 bp product (spanning region 72,653,763–72,653,985) which gives two fragments of 121 bp and 102 bp after digestion with SexAI only in its WT form, but not if mutated to *Nedd4*^*C854A*^. Correctly targeted mice show a product of 223 bp due to the loss of the SexAI site. Mice devoid of additional undesired mutations were used for colony propagation.

*Rorc*^*Y479F*^ mice are genotyped by PCR amplifying a 252 bp PCR product (spanning region 94,304,452–94,304,703) from 2 WT C57/Bl6Tac controls and from 3 male potential founders were digested with DraI and sequenced. WT product is not digested by Dral. Correctly targeted mice show 158 bp and 94 bp fragments following DraI digestion. Mice devoid of additional undesired mutations were used for colony propagation.

### Naïve CD4^+^CD25^−^ T cells isolation and in vitro differentiation of Th1, Th2, Th17, and inducible regulatory T cells (iTregs)

Naïve CD4^+^CD25^−^ T cells from *Nedd4*^*CreER*^ mice pretreated with tamoxifen or corn oil were isolated by the Naive CD4^+^ T Cell Isolation Kit (Miltenyi Biotec). The naive CD4^+^ T cells were stimulated by plate-coated anti-CD3 (5 µg/ml) and anti-CD28 (1 µg/ml) in the presence of Th1, Th2, Th17 (non-pathogenic and pathogenic), and iTregs as described previously [23, 24]: recombinant mouse IL-2 (rmIL-2) (10 ng/ml), IL-12 (20 ng/ml), and anti-IL-4 (10 µg/ml) for Th1 condition; IL-4 (50 ng/ml), anti-IFN-γ (10 µg/ml), anti-IL-12 (10 µg/ml) for Th2 condition; mIL-6 (25 ng/ml), TGF-β (2 ng/ml), anti-IFN-γ (5 µg/ml), anti-IL-4 (3 µg/ml) for non-pathogenic Th17 condition; mIL-6 (25 ng/ml), IL-1β (20 ng/ml), IL-23 (20 ng/ml), anti-IFN-γ (5 µg/ml), and anti-IL-4 (3 µg/ml) for pathogenic Th17 condition; rmIL-2 (10 ng/ml), TGF-β (2 ng/ml), anti-IFN-γ (10 µg/ml), and anti-IL-4 (10 µg/ml) for iTreg condition. On day 4 to 7, harvest the cells and wash with 3 volumes of fresh RPMI medium. For Th1, Th2 and Th17 subsets, the cells were restimulated with 50 ng/ml PMA and 750 ng/ml ionomycin for 4 h. Then the cells were stained in different combinations of fluorochrome-conjugated antibodies as mentioned previously. For the identification of iTregs, the cells were surface stained with FITC-conjugated anti-mouse CD4 (Cat. 11–0042; eBioscience), APC-conjugated anti-CD25 (Cat. 17–0251; eBioscience), then fixed, and permeabilized using the Foxp3/transcription factor staining buffer (Cat 00–5523; eBioscience) and subsequently stained with 0.5 µg of PE-conjugated Rat IgG2a K isotype control (Cat. 12–4321; eBioscience) or PE-conjugated anti-mouse Foxp3 (7,205,775–40; eBioscience).

### Detection of Th17 cells in the lamina propria of intestinal tissues

The Lamina Propria lymphocytes (LPLs) were isolated as previously described [11]. In brief, *Nedd4*^*CreER*^ mice pretreated with corn oil or tamoxifen were euthanized, and intestines were removed and placed in ice-cold PBS. After removal of residual mesenteric fat tissue, and Peyer’s patches, the intestines were cut into 1.5 cm pieces, and incubated twice with 5 mM EDTA in HBSS for 15–20 min at 37 ℃. The epithelial cell layer, which contains intraepithelial lymphocytes (IELs), was removed by passing through a 100 µM cell strainer. The pieces were then further cut into 1 mm^2^ pieces, and digested with Collagenase D (0.5 mg/ml), 4% FBS, and DNAase (0.5 mg/ml), and 50 U/ml Dispase. The solution was passed through a 40 mm cell strainer. The pieces were collected and placed into fresh digestion solution and this process was repeated for three times. The supernatants from all three digestions were combined together, washed once with cold PBS, resuspended in 10 ml of the 40% fraction of a 40:80 Percoll gradient. After Percoll gradient separation, LPLs were collected at the interphase of the Percoll gradient, washed once, and resuspended in FACS buffer. LPLs of *Nedd4*^*CreER*^ mice pretreated with corn oil or tamoxifen were stimulated with PMA/ionomycin for 5 h. TCRβ^+^CD4^+^ IL-17^+^ T cells were determined by flow cytometry.

### EAE induction

Various groups of mice at 8–12 weeks were subcutaneously injected (s.c.) over four sites in the flank with 100 µl of emulsified CFA supplemented with 200 μg of MOG_33–55_ in an emulsion with IFA, which contain 500 μg of heat-inactivated M. tuberculosis (Difco). 300 ng pertussis toxin (List) per mouse in PBS was injected i.p. at the time of immunization and 48 h later. The mice were evaluated daily for clinical signs of EAE. Mice were scored on scale of 0 to 5 [25, 26]: 0, no clinical disease; 1, limp/flaccid tail; 2, moderate hind limb weakness; 3, severe hind limb weakness; 4, complete hind limb paralysis; and 5, quadriplegia or premoribund state.

### Ex vivo MOG_35-55_-specific CD4^+^ T cell recall responses

dLN cells from MOG_35–55_-immunized *Cd4 Cre-Nedd4*^*f/f*^ and *Cd4 Cre* mice (7 days after immunization) were labeled with CFSE and cultured with 20 μg/ml MOG_35–55_ for 5 days. The proliferation rates of CD4^+^ T cells were determined by CFSE dilution. The supernatants collected from these cultures were subjected for ELISA for IL-17A, IL-17F, IL-1β, IL-6, IL-21, GM-CSF, IL-10 and IFN-γ using the sandwich ELISA kits (BioLegend). All the procedures were performed according to the manufacturer’s instructions.

### Detection of in vivo Th1 and Th17 responses during EAE induction

For detection of T cell compartments in *Nedd4*^*CreER*^ mice pretreated with oil or tamoxifen, cells were stained in different combinations of fluorochrome-conjugated antibodies. For detection of Th1 and Th17 responses, *Cd4 Cre* and *Cd4 Cre-Nedd4*^*f/f*^ mice were immunized with MOG_35-55_ in CFA. The dLN cells were collected at day 7 after immunization, and stimulated with 20 µg/ml MOG_35-55_ in the culture medium for 3 days. The cells were restimulated with 50 ng/ml PMA and 750 ng/ml ionomycin for 4 h in the presence of Golgi-stop, surface-stained with anti-CD4, and intracellularly stained with anti-IFN-γ or anti-IL-17, respectively. Treg cells were detected by using Mouse Regulatory T Cell Staining Kit (eBioscience) according to the instructions. To determine MOG_35–55 -_specific Th17 response, the dLN cells from the above culture were surface-stained with anti-CD4 and PE-conjugated I-A^b^-MOG_35-55_ tetramer, and intracellularly stained with anti-IL-17. Flow cytometry data were acquired on a FACSCanto™ II, LSR™ II (both BD Bioscience, San Jose, CA, USA. FlowJo (Treestar, Ashland, OR, USA) software was used for analysis.

### Isolation of central nervous system (CNS) infiltrating cells

Spinal cord was removed by insufflation using 20 ml 4 ℃ RPMI expressed through a 19G needle. The spinal cord was transferred to 10 ml fresh, cold RPMI and homogenized by using the 5 ml syringe. Cells were pelleted by centrifugation at 300 ×*g* for 5 min at 4 ℃ and the supernatant was discarded. The cells were resuspended in 1 ml digestion solution (PBS with Calcium and Magnesium, 1.5 mg/ml Collagenase II, 50 μg/ml DNase I) by using vortexer and incubated for 30 min, 37 ℃ in water bath. 30–70% colloid Percoll was used to isolate CNS infiltrating cells. The cells were carefully harvested from the interphase 30%-70% Percoll fraction and were transferred into new 15 ml Falcon tube containing 8 ml PBS/FCS. Cells were pelleted by centrifugation at 900 ×*g*, 5 min, 4 ℃ and the supernatant was discarded. The isolated cells were further used for detection of Th1 and Th17 responses by flow cytometry as previously described [25, 26].

### Immunoprecipitation and Western blotting

For co-immunoprecipitation assays, naïve CD4^+^ T cells were differentiated into Th17 cells in vitro. Cells were lysed in 0.5% NP40 lysis buffer. The cell lysates were immunoprecipitated with anti-RORγt conjugated to agarose, and blotted with anti-Nedd4 (1:1000), anti-Itch (1:1000) and anti-Nedd4-2 (1:1000). To detect RORγt ubiquitination, naïve CD4^+^ T cells from *Nedd4*^*CreER*^ mice pretreated with tamoxifen or corn oil were cultured under Th17 condition for 72 h. The cells were restimulated with anti-CD3 and anti-CD28 for various times, and then lysed in RIPA buffer containing 2% SDS, sonicated, and heated to 95 °C for 5 min, then diluted to 0.5% of SDS without SDS before immunoprecipitation in order to disrupt the proteins associated with RORγt. The cell lysates were immunoprecipitated with anti-RORγt (2 µg/ml), and blotted with anti-ubiquitin (1:1000), or with anti-K27-specific ubiquitin antibodies (1:1000). To assess the association between RORγt and the different domains of Nedd4, HEK293T cells were transfected with Flag-tagged Nedd4 or its truncated fragments, together with Myc-tagged RORγt, and lysed in 0.5% NP40 lysis buffer. The cell lysates were immunoprecipitated with anti-Myc and blotted with anti-Flag. To assess the ubiquitination type of RORγt, HEK293T cells were transfected with HA-tagged ubiquitin or ubiquitin mutants, together with Myc-tagged RORγt and Flag-tagged Nedd4, and lysed RIPA buffer containing SDS with a denaturing step. The cell lysates were immunoprecipitated with anti-Myc (2 µg/ml), and blotted with anti-HA (1:1000). To confirm whether K112 is really RORγt ubiquitination site, we transfected HEK293T cells with Flag-tagged Nedd4, Myc-tagged RORγt or RORγt K112R, and HA-tagged ubiquitin. The cell lysates were immunoprecipitated with anti-Myc (2 µg/ml), and blotted with anti-HA (1:1000). To determine the expression of RORγt during the induction of EAE, WT and *Nedd4*^*C854A*^ mice were immunized with MOG_35-55_ in CFA for 3, 5, and 7 days. CD4^+^ T cells from dLNs from each time point were isolated, lysed, and analysed by immunoblotting with anti-RORγt. To assess whether Nedd4 facilitates RORγt protein synthesis, we cultured naïve CD4^+^ T cells from WT and *Nedd4*^*C854A*^ mice under the pathogenic Th17 condition in the presence or absence of cycloheximide (CHX; 20 µM) for 1, 2, and 3 days, lysed. The cell lysates were blotted with anti-RORγt antibody.

### GST pull-down assay

GST pull-down assays were performed using standard procedures. To express various GST-fused Nedd4 proteins, plasmids were constructed using a PCR-based method with vector pGEX-4 T-1 (GE Healthcare). For the GST pull-down assay, ~ 10 μg GST fusion proteins were mixed with ~ 10 μg or the indicated amount of Nedd4 fusion proteins. The mixtures were incubated at 4 ℃ for 1 h with gentle shaking, and then 50 μl pre-rinsed glutathione sepharose beads (GE Healthcare) was added, followed by incubation at 4 ℃ for another 1 h. The beads were then washed 5 times with 1 × TBST buffer. Finally, 1 × SDS sample-loading buffer was added to the beads, and the mixture boiled for 5 min, prior to SDS-PAGE analysis.

### Plasmids and transfection

Flag-tagged Nedd4 and Nedd4 C867A plasmids [27] were provided by Dr. Nobuyuki Tanaka (Miyagi Cancer Center Research Institute, Miyagi, Japan). RORγt reporter plasmid, RORγt and RORγt Y479F plasmids [28] were gifts from Dr. Zuoming Sun (City of Hope, CA) in which the Myc tag was inserted at the Mutagenex Inc. (Suwanee, GA, USA). HA-tagged ubiquitin plasmids were obtained from Dr. Zhijian Chen (University of Texas at Southwestern University, Dallas, TX, USA). The truncated fragments of Nedd4 plasmids, RORγt K112R mutant, and ubiquitin mutant plasmids were generated by the Mutagenex Inc. (Suwanee, GA, USA). HEK293T cells were transfected with various plasmids by calcium precipitation [23]. For reconstitution experiments, naive CD4^+^ T cells from *Rorc*^–/–^ mice were reconstituted with Myc-tagged RORγt or Myc-tagged RORγt K112R by nucleofection (Lonza 4D-Nucelofector) and cultured for 24 h. 24 h later, cells were restimulated with anti-CD3 and anti-CD28 for 15 min, and then lysed in 2% RIPA lysis buffer for RORγt ubiquitination assay.

### RORγt luciferase reporter assay

RORγt reporter assay was performed according to the protocol published previously [28]. In brief, HEK293T cells were maintained in 24-well plates (2 × 10^5^ in each well) in Dulbecco’s modified Eagle medium supplemented with 10% FBS, 2 mM glutamine, 100 U/ml penicillin, and 100 μg/ml streptomycin. At ~ 80% confluence, cells were transfected with RORγt reporter plasmid (100 ng), pSV40-Renilla luciferase vector (50 ng), and expression vectors (Flag-tagged Nedd4 or Nedd4 C867A, together with HA-tagged ubiquitin, or Flag-tagged Nedd4 together with HA-tagged K27 ubiquitin or K27R ubiquitin; 0.5 μg) by calcium precipitation. Cells were collected after 24 h and lysed in 20 μl Passive Lysis Buffer; activities were measured by the Dual Luciferase system, according to the manufacturer’s instructions (Promega, WI, USA), and normalized against Renilla luciferase activities. “Folds of stimulation” represents normalized luciferase activity divided by the result of reporter-only groups.

### Electromobility superswift assay (EMSA)

For EMSA assay, naïve CD4^+^ T cells from *Cd4 Cre* and *Cd4 Cre-Nedd4*^*f/f*^ mice were differentiated under Th17 differentiation condition for 72 h in vitro and re-stimulated by anti-CD3 and anti-CD28 in different time points. The nuclear fraction protein of the cells were generated as previously described [29]. In brief, the cells were triturated with ice-cold 0.5% NP-40 containing buffer A (0.5%NP-40, 10 mM HEPES, 10 mM KCl, 1.5 mM MgCl_2_, 0.5 mM DTT, 1 mM PMSF, 10 µg/ml Apoptonin and 10 µg/ml Leupeptin). The cells were centrifuged at 8000 *g* for 30 s at 4 °C, the supernatant was discarded. The pellets were resuspended with ice-cold buffer C (20 mM HEPES, 420 mM NaCl, 0.2 mM EDTA, 1.5 mM MgCl_2_, 0.5 mM DTT and 25% Glycerol) and sonicated using microprobes on ice, and centrifuged at 8000 *g* for 30 s at 4 °C. The nuclear extracts of Th17 cells were incubated with a biotin-labeled ROR element (RORE) in *Il17 CNS2* (CNS2), 5^′^-GAAAGTTTTCTGACCCACTTTAAATCAATTT-3′. The protein/DNA complexes were separated on a nondenaturing polyacrylamide gel, transferred to a nylon membrane, and detected using Streptavidin-HRP-conjugated chemiluminescent substrate.

### Mass spectrometry

Gel-assisted Isolation: To identify the potential ubiquitination site(s) of RORγt mediated by Nedd4, we transfected HEK293T cells with Flag-tagged Nedd4, Myc-tagged RORγt, and HA-tagged ubiquitin. The cells were collected and washed with PBS and then lysed in RIPA buffer containing 2% SDS, which were then diluted to 0.2% of SDS. The cell lysates were immunoprecipitated with anti-RORγt conjugated to Protein A agarose (GE Health Care). The beads were then washed 5 times with cold PBS. 1 × SDS sample-loading buffer was added to the beads, and the mixture boiled for 5 min. The gel was stained with Silver Stain kit (Pierce Biotechnology), and the bands corresponding to RORγt and above were excised. The gel pieces were further treated with ACN, to effectively “dry” the gel segments and then reduced in 50 μl of 10 mM DTT at 56 ℃ for 60 min. After this, gel-trapped proteins were alkylated with 55 mM Chloroacetamide (CAM) for 30 min at room temperature. The gel pieces were washed and incubated with trypsin at 37 ℃ for 16 h. The combined peptide extracts were lyophilized and resuspended in 15 μl of LC buffer A and analyzed in conjunction with liquid chromatography (LC) tandem mass spectrometry (MS/MS) on the orbitrap.

LC–MS/MS: Mass spectrometry data were collected using an Orbitrap Fusion Lumos mass spectrometer (Thermo Fisher Scientific, San Jose, CA) coupled to an Easy-nLC-1200™ System (Proxeon P/N LC1400). Typically, 3–4 μl of reconstituted digest is loaded on a 2.5 cm C18 trap (New Objective, P/N IT100-25H002) coupled to an analytical column through a microcross assembly (IDEX, P/N UH-752). Peptides are desalted on the trap using 16 μl mobile phase A in 4 min. The waste valve is then blocked and a gradient begins flowing at 0.4 μl/min through a self-packed analytical column; 10 cm in length x 75 μm id. Peptides were separated in-line with the mass spectrometer using a 70 min gradient composed of linear and static segments wherein buffer A is 0.1% formic acid and B is 95% ACN, 0.1% Formic acid. The gradient begins first holds at 4% for 3 min then makes the following transitions (%B, min): (2, 0), (35, 46), (60, 56), (98, 62), (98, 70).

Tandem mass spectrometry on the LUMOS Orbitrap: Data acquisitions begin with a survey scan (m/z 380 -1800) acquired on an Orbitrap Fusion Lumos mass spectrometer (Thermo) at a resolution of 120,000 in the off axis Orbitrap segment (MS1) with Automatic Gain Control (AGC) set to 3E06 and a maximum injection time of 50 ms. MS1 scans were acquired every 3 s during the 70 min gradient described above. The most abundant precursors were selected among 2–6 charge state ions at a 1E05 AGC and 70 ms maximum injection time. Ions were isolated with a 1.6 Th window using the multi segment quadrupole and subject to dynamic exclusion for 30 s if they were targeted twice in the prior 30 s. Selected ions were then sequentially subjected to both CID and HCD activation conditions in the IT and the ion routing multipole respectively (IRM). The AGC target for CID was 4.0E04, 35% collision energy, with an activation Q of 0.25 and a 75 ms maximum fill time. Targeted precursors were also fragmented by high energy collision-induced dissociation (HCD) at 30% collision energy in the IRM. HCD fragment ions were analyzed using the Orbitrap (AGC 1.2E05, maximum injection time 110 ms, and resolution set to 30,000 at 400 Th). Both MS2 channels were recorded as centroid and the MS1 survey scans were recorded in profile mode.

Proteomic Searches: Initial spectral searches were performed with both Mascot version 2.6.2 (MatrixScience) and Byonic search engines (Protein Metrics ver. 2.8.2). Search databases were composed of the Uniprot KB for species 9606 (Human) downloaded 10/24/2017 containing 92,645 sequences and Uniprot KB for taxonomy 562 (E. coli) downloaded on 11/08/2017 containing 10,079 sequences. For Byonic searches, these two data bases were directly concatenated. In either search an equal number of decoy entries were created and searched simultaneously by reversing the original entries in the target databases. Precursor mass tolerance was set to 10 ppm and fragments acquired in the Linear Trap and the Orbitrap were searched at 0.4 Da and 10 ppm respectively. A fixed 57 Da modification was assumed for cysteine residues while a variable Oxidation modification was allowed at methionine. A variable GG modification at lysine was set to monitor ubiquitination with potential phosphorylation accessed at Ser and Thr residues. The False Discovery Rate was maintained at 1% by tracking matches to the decoy database.

Both Mascot and Byonic search results were combined and validated using Scaffold ver. 4.8.5 (Proteome Software). Protein assignments required a minimum of two peptides established at 70% probability (Local FDR algorithm) and an overall 95% protein probability (assigned by Protein Prophet). Approximately 300 protein families (including common contaminants) were assigned at a total FDR to 1.2%. Proteins were annotated with GO terms from goa_uniprot_all.gaf downloaded on May 3, 2017.

### Lentivirus-mediated gene transfer

Lentivirus-mediated gene transfer was performed as described previously [23]. HEK293T were transiently co-transfected with pRV-3 GFP encoding GFP tagged RORγt or RORγt K112R expression vectors together with (pHEP and pENV) packaging plasmids containing vesicular stomatitis virus G protein (VSV-G) by using calcium precipitation. Lentivirus-containing medium, 48 h after transfection, was collected and supplemented with 8 mg/ml of Polybrene (Santa Cruz). *Rorc*^–/–^ CD4^+^ T cells were infected by replacing the cell culture medium with the viral supernatant, and were stimulated with anti-CD3 (2 μg/ml) and anti-CD28 (1 μg/ml) for 24 h or were differentiated under Th17 condition for 3 days.

### Expression of NEDD4, NEDD4-2, and RORγT in CD4^+^ T cells from MS patients

CD4^+^ T cells were isolated from 6 MS patients (treatment-naïve and in remission) and 6 age- and sex-matched healthy controls by a Human CD4^+^ T cell isolation kit, and lysed in RIPA buffer. The cell lysates were blotted with antibodies against NEDD4, NEDD4-2, and RORγt, respectively. For ex vivo flow cytometric analysis of NEDD4 and RORγt expression, human PBMC from 6 MS patients (treatment-naïve and in remission) and 13 healthy controls were surface stained with AlexaFlour® 700 anti-CD3 (557,943, BD Biosciences), PE-conjugated anti-CD4 (555,347, BD Biosciences) and PacificBlue-conjugated anti-CD45RO (304,216, BioLegend) followed by intracellular staining with anti-NEDD4 (H-135, sc-25508) and APC conjugated anti-RORγT (17–6988-82, eBioscience) followed by Alexa Flour^®^ 488 conjugated anti-rabbit IgG (A21206, Invitrogen).

### Silencing *NEDD4* gene in human CD4^+^ T cells, and human T_H_17 differentiation assay

For in vitro human T_H_17 differentiation assay, naïve CD4^+^ T cells were isolated from PBMCs using a human CD4^+^ T cell isolation kit (Miltenyi Biotech 130–094-131) and resuspended at 1 × 10^6^ cells/ml in Xvivo15 serum-free media (04-418Q, Lonza), followed by adding *Accell NEDD4* siRNA (2 µg/ml) (4734, Horizon) or control siRNA using Amaxa Human T cell Nucleofector kit (VPA-1002, Lonza) according to manufacturer’s manual. After nucleofection, cells were rest in RPMI-1640 containing 10% FCS for 6 h before polarizing in Th17 differentiation conditions: anti-IL-4 (7 μg/ml) (554,481, BD Biosciences), anti-IFN-γ (7 μg/ml) (554,698, BD Biosciences), IL-1β (50 ng/ml) (554,602, BD Biosciences), IL-6 (30 ng/ml) (550,071, BD Biosciences) and IL-23 (50 ng/ml) (574,106, BioLegend), and in the presence of anti-CD3 (0.5 μg/ml) (16–0037-85, eBioscience) and anti-CD28 (0.5 μg/ml) (16–0289-85, eBioscience) and incubated for 5 days at 37 °C. At day 5, cells were washed and stained with BV421 conjugated anti CD4 (566,970, BD Biosciences), PE conjugated anti CXCR3 (557,185, BD Biosciences) and PE-CY7 conjugated anti CCR6 (560,620, BD Biosciences).

### Sorting of pathogenic Th17 cells from MS patients

Fresh peripheral blood samples were collected from de-identified patients with relapsing–remitting multiple sclerosis (RRMS). PBMCs were isolated using density gradient centrifugation. Pathogenic Th17 (CCR6^+^CXCR3^+^) cells were sorted from PBMCs using a BD FACSAriaTM III cell sorter. Following sorting, the cells were amplified by stimulation with anti-CD3/CD28 Dynabeads for 7 days.

### Molecular docking analysis

The structures of NEDD4 (AF-P46935-F1) and RORG (AF-P51450-F1) were obtained from AlphaFold Protein Structure Database. Molecular docking simulations were carried out using GARMM to determine the interactions between NEDD4 and RORG. All docking results were visualized by Pymol.

### Statistical analysis

GraphPad Prism Software (San Diego, CA, USA) was utilized for statistical analysis. Error bars represent standard deviation. A two-tailed Student's t-test was applied for statistical comparison of two groups or, where appropriate and a Mann–Whitney U test for nonparametric data (EAE scoring). Differences were considered significant at p < 0.05. No animals were excluded from the analysis. Pearson correlation coefficient was used to determine a linear correlation between NEDD4 and RORγt expression in human heathy controls and MS patients. Mice were allocated to experimental groups based on their genotypes and were randomized within their sex- and age-matched groups. No statistical method was used to predetermine sample size. It was assumed that normal variance occurs between the experimental groups.

## Results

### Nedd4 Is specifically required for Th17 cell differentiation in vitro and in vivo

The studies on Nedd4 at the cellular and biochemical levels have been hindered by the embryonic lethality of conventional Nedd4^–/–^ mice. Previously studies have shown that Nedd4 positively regulates T cell activation in Nedd4^+/+^ and Nedd4^–/–^ fetal liver chimeras [20, 21], but whether Nedd4 also regulates T helper (Th) cell differentiation is unknown. To overcome this caveat, we generated a conditional *Nedd4* floxed allele by targeted disruption of the *mNedd4* gene by the *Cre-loxP* strategy [22]. We have crossed mice bearing LoxP-flanked alleles encoding Nedd4 with *Rosa26-Cre-ERT2* mice (in which sequence encoding a fusion of Cre recombinase and the estrogen receptor (ER) was recombined into the ubiquitously expressed Rosa26 locus) to generate *Nedd4*^*fl/fl*^*Rosa26-Cre-ERT2* mice (called '*Nedd4*^*CreER*^ mice' here, Supplementary Fig. 1A). We also crossed mice bearing LoxP-flanked alleles encoding Nedd4 with *Cd4 Cre*, *LysM Cre*, *Cd11c Cre* and *Cd19 Cre* mice to generate *Cd4 Cre-Nedd4*^*f/f*^, *LysM Cre-Nedd4*^*f/f*^, *Cd11c Cre-Nedd4*^*f/f*^*,* and *Cd19 Cre-Nedd4*^*f/f*^ mice, respectively, to specifically delete Nedd4 in T cells, myeloid cells, DCs, and B cells, respectively (Supplementary Fig. 1B).

The phenotypic analysis of mice lacking Nedd4 in T cells showed that there was no significant alteration in composition in T cell compartments. These included distributions of CD4^+^ single positive (SP), CD8^+^ SP, CD4^+^CD8^+^ double positive (DP), CD3^+^CD4^–^CD8^–^ double negative (DN) thymocytes, expression of TCRβ in CD4^+^ SP and CD8^+^ SP thymocytes, distributions of splenic CD4^+^, CD8^+^ T cells, and their expression of activation/memory markers CD69, CD25, CD62L and CD44, as well as CD4^+^CD25^+^Foxp3^+^ regulatory T cells (Tregs) between mice deficient or sufficient for Nedd4 (Supplementary Fig. 1C–I), which is consistent with previous reports[20, 21]. Note that the suppressive activity of Tregs of mice lacking Nedd4 was not altered (Supplementary Fig. 1J).

**Fig. 1:**
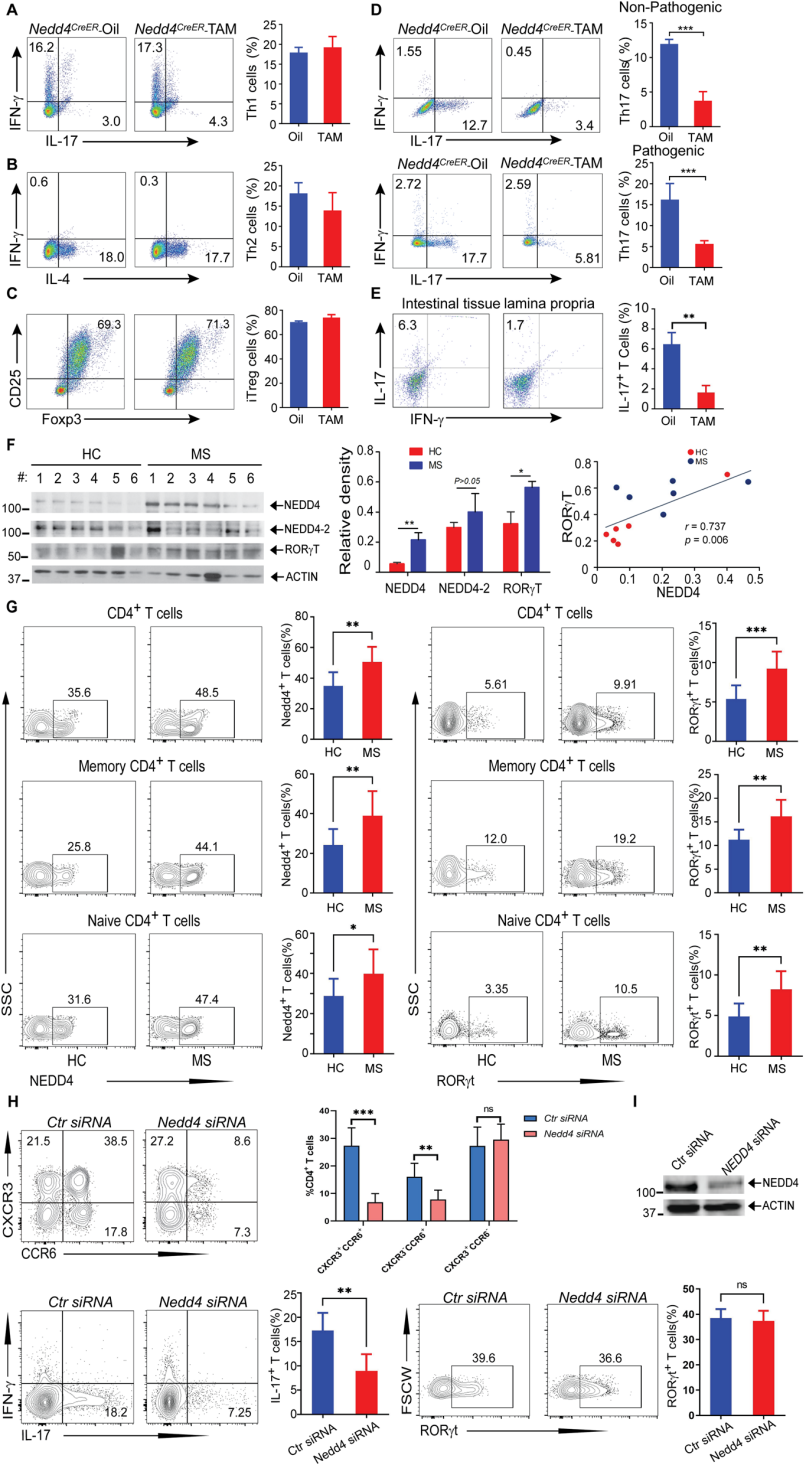
Nedd4 potentiates Th17 cell differentiation in vitro and in vivo. **A**–**C** Th1, Th2, and iTreg cell differentiation assays using naïve CD4^+^ T cells from *Nedd4*^*CreER*^ mice pretreated with tamoxifen or corn oil (n = 3 mice per group). **D** Pathogenic vs. non-pathogenic Th17 cell differentiation assays using naïve CD4^+^ T cells from in *Nedd4*^*CreER*^ mice pretreated with tamoxifen or corn oil (n = 3 mice per group). ***p < 0.001; student *t* test. **E** Th1 and Th17 cells in the lamina propria of *Nedd4*^*CreER*^ mice small intestinal tissue pretreated with tamoxifen or corn oil (n = 3 mice per group). **p < 0.01; student *t* test. **F** Immunoblot analysis of NEDD4, NEDD4-2, and RORγT in CD4^+^ T cells from MS patients (n = 6) and age- and sex-matched healthy controls (n = 6). *p < 0.05, **p < 0.01; student *t* test. *r* = 0.737, Pearson correlation coefficient. The relationship between two variables is generally considered strong when their r value is larger than 0.7. **G** Expression of NEDD4 and RORγt ex vivo in total CD4^+^ T cells, vs. naïve and memory CD4 + T cells from MS patients and healthy controls (HC) by flow cytometry. Cumulative data from multiple donor samples (10 MS patients and 13 healthy donors). *p < 0.05, **p < 0.01, ***p < 0.001; unpaired student *t* test. **H** Flow cytometry evaluating Th17 cell differentiation of human naive CD4^+^ T cells from six healthy blood donors nucleofected with *Ctr siRNA* or *Nedd4 siRNA* at the temporal peak of differentiation conditions. **p < 0.01, ***p < 0.001; paired student *t* test. **I** NEDD4 knock down efficiency was determined by immunoblot analysis of lysates of T cells transfected with NEDD4 siRNA or control siRNA on day 3

To address whether Nedd4 regulates Th cell differentiation, we performed in vitro Th1, Th2, Th17 (TGF-β + IL-6 for non-pathogenic Th17; IL-1β, IL-6, and IL-23 for pathogenic Th17) [8, 9], and inducible Treg (iTreg) differentiation assays [24] using naïve CD4^+^CD25^–^ T cells from *Nedd4*^*CreER*^ mice pretreated with tamoxifen or vehicle corn oil. As shown in Fig. 1A–D, differentiation of non-pathogenic and pathogenic Th17, but not Th1, Th2, and iTreg cells, was significantly reduced in mice lacking Nedd4. The lamina propria of intestinal tissue is a highly active lymphoid microenvironment and is a reservoir for Th17 cells in vivo [11, 30]. Consistent with our in vitro data, CD4^+^IL-17^+^ T cells in the lamina propria were reduced in *Nedd4*^*CreER*^ mice pretreated with tamoxifen compared to those pretreated with corn oil (Fig. 1E). Since the activation of CD4^+^ T cells will influence its differentiation, we employed *Cd4 Cre-Nedd4*^*f/f*^ mice to determine whether the knockout of Nedd4 affects the activation of CD4^+^ T cells. As shown in Supplementary Fig. 2A, Nedd4 deficiency did not affect the activation of naive CD4 + T cells after stimulating with CD3 and CD28 for 24 h. Similarly, we found that there was no significant change in Nedd4 expression after T cell activation (Supplementary Fig. 2B).

To further assess whether the ability of Nedd4 to positively regulate T cell development plays a role in MS pathogenesis, we first measured NEDD4, NEDD4-2, and RORγt protein expression in CD4^+^ T cells from 6 relapsing–remitting multiple sclerosis (RRMS) patients (treatment-naïve and in remission) and 6 age- and sex-matched healthy donors. We found that NEDD4 and RORγT expression was significantly increased in MS T cells compared to CD4^+^ T cells from the healthy controls by Western blotting (Fig. 1F). We did not observe any significant increase in NEDD4-2 expression in CD4^+^ T cells from MS patients (Fig. 1F). To confirm these data, we measured the expression of NEDD4 and RORγt in CD4^+^ T cells from 13 healthy donors and 10 MS patients by flow cytometry. As shown in the left panel of Fig. 1G, NEDD4 expression was significantly augmented in both naive and memory CD4^+^ T cells from MS patients than healthy donors. Consistent with this data, RORγT expression was significantly enhanced in both naive and memory CD4^+^ T cells from MS patients than healthy donors (Fig. 1G, right panel).

We conducted experiments to investigate the impact of NEDD4 on mouse Th cells and iTreg differentiation, revealing a significant influence on the differentiation of both non-pathogenic and pathogenic Th17 cells in Nedd4-deficient mice. To assess the conservation of this effect, analogous experiments were performed using human CD4 + T cells. We employed a SMARTpool siRNA system (Accell siRNA, Dharmacon) comprising of four distinct sequences targeting the same gene to minimize off-target effects. Human Th1, Th17, and pathogenic Th17 cells were characterized based on the surface markers CCR6 and CXCR3: CCR6^–^CXCR3^+^ (Th1), CCR6^+^CXCR3^–^ (Th17), and CCR6^+^CXCR3^+^ (pathogenic Th17) subsets [31, 32]. Following transfection with *Ctr siRNA* or *Nedd4 siRNA*, human naive CD4^+^ T cells were placed in Th17 polarizing conditions (Fig. 1H). In support of our data obtained from mice lacking Nedd4, silencing NEDD4 gene in human CD4^+^ T cells (Fig. 1J) markedly inhibited the proliferation of CCR6 ^+^ CXCR3^–^ and CCR6^+^CXCR3^+^ subsets (Fig. 1H, upper panel). Consistent with this result, silencing Nedd4 resulted in a marked reduction in the expression of IL17 by naive CD4^+^ T cells in Th17 differentiation conditions, while the expression of RORγt was not significantly changed (Fig. 1H, lower panel), which suggests that the regulation of Th17 cells by Nedd4 is not achieved by altering the expression level of RORγt but by modulating the function of RORγt. The above findings suggest that the NEDD4/RORγT axis is possibly involved in MS pathogenesis by facilitating Th17 responses.

### Mice deficient for Nedd4 or Nedd4 in T cells developed ameliorated EAE and display an impaired antigen-specific Th17 response

**Fig. 2:**
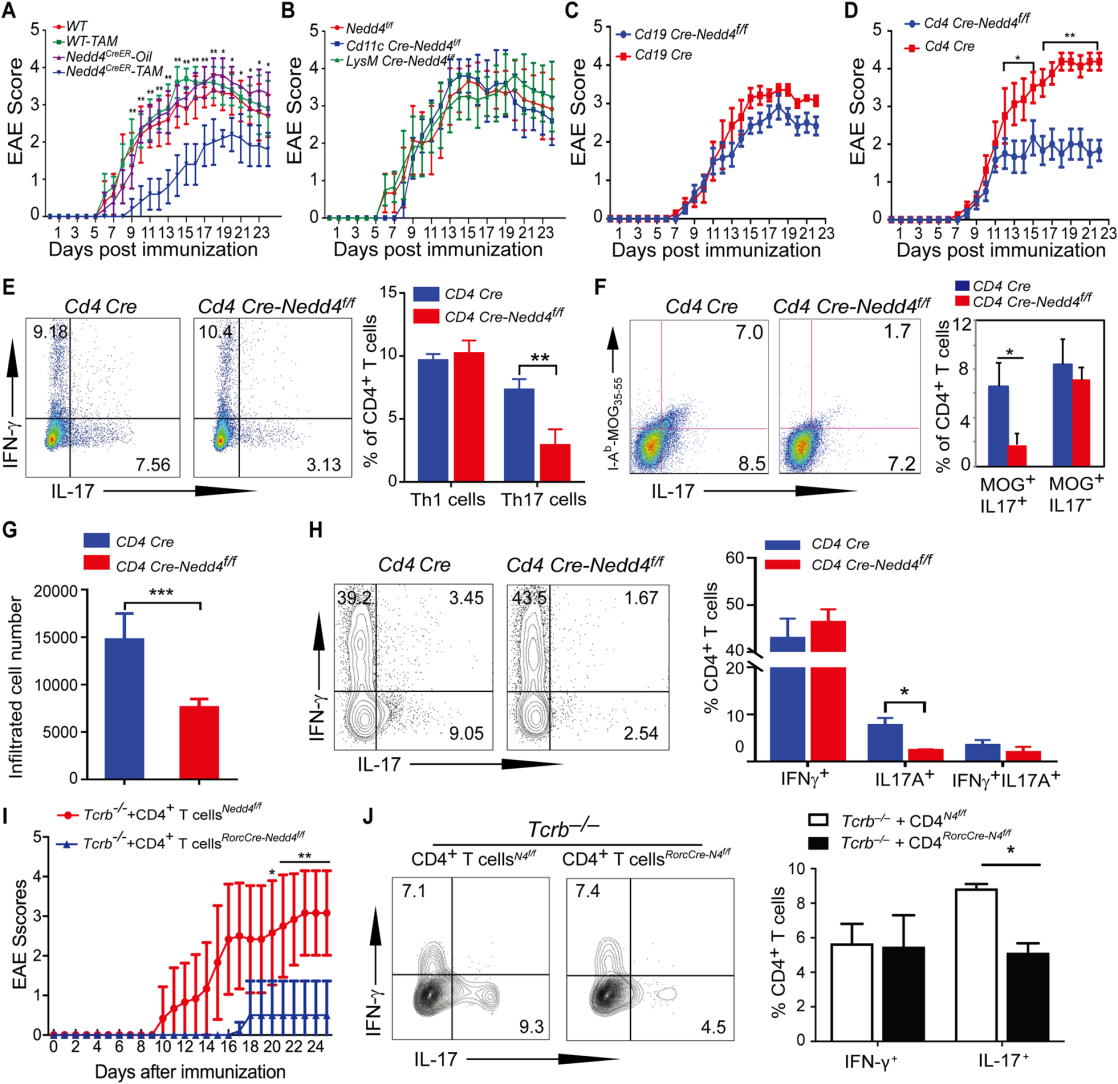
Nedd4 Expression in T cells confers the susceptibility of mice to EAE induction and is required for antigen-specific Th17 response. EAE was induced by MOG_35-55_ in CFA, and the disease activity was monitored for 21–25 days after immunization. For ex vivo T cell responses, *Cd4 Cre* and *Cd4 Cre-Nedd4*^*f/f*^ mice, or *Tcrb*^–/–^ mice adoptively transferred with naïve CD4^+^ T cells from *Nedd4*^*f/f*^* and Rorc Cre*-*Nedd4*^f/f^ mice were immunized with MOG_35-55_ in CFA, and mice were analyzed at day 8 after immunization for Th1/Th17 in draining lymph nodes (dLNs) and at day 14–16 for total infiltrating cells and Th1/Th17 in the spinal cords. **A** EAE scores of *Nedd4*^*CreER*^ mice pretreated with tamoxifen or corn oil, or B6 mice (n = 5 mice per group) pretreated with or without tamoxifen. WT, WT-TAM, or *Nedd4*^*CreER−oil*^ vs. *Nedd4*^*CreER−TAM*^. *p < 0.05, **p < 0.01; Mann–Whitney *U* test. **B** EAE scores of *Nedd4*^*f/f*^ (n = 6), *Cd11c Cre-Nedd4*^*f/f*^ (n = 5), *LysM Cre-Nedd4*^*f/f*^ (n = 6) mice. **C** EAE scores of *Cd19 Cre* (n = 7) and *Cd19 Cre-Nedd4*^*f/f*^ (n = 6) mice. **D** EAE scores of *Cd4 Cre* (n = 8) and *Cd4 Cre-Nedd4*^*f/f*^ (n = 5) mice. *p < 0.05, **p < 0.01; Mann–Whitney *U* test. **E** Flow cytometric analysis of CD4^+^IFN-γ^+^ Th1 cells and CD4^+^IL-17^+^ Th17 cells of *Cd4 Cre* and *Cd4 Cre-Nedd4*^*f/f*^ mice (n = 3 per group) at day 8 after immunization. **p < 0.01; student *t* test. **F** Flow cytometric analysis of I-A^b^-MOG_35-55_^+^IL-17^+^ T cells of *Cd4 Cre* and *Cd4 Cre-Nedd4*^*f/f*^ mice (n = 3 mice per group) at day 8 after immunization. *p < 0.05; student *t* test. **G** Numbers of infiltrating cells in CNS from *Cd4 Cre* and *Cd4 Cre-Nedd4*^*f/f*^ mice (n = 3 mice per group) at day 14–16 after immunization. ***p < 0.001, student *t* test. **H** Flow cytometric analysis of CD4^+^IFN-γ^+^ Th1 cells and CD4^+^IL-17^+^ Th17 cells in spinal cords of *Cd4 Cre* and *Cd4 Cre-Nedd4*^*f/f*^ mice (n = 3 per group). *p < 0.05, student *t* test. **I** EAE scores of *Tcrb*^–/–^ mice (n = 7 mice per group) adoptively transferred with CD4^+^ T cells from *Nedd4*^*f/f*^* and Rorc Cre*-*Nedd4*^f/f^ mice upon immunization with MOG_35-55_ in CFA. *p < 0.05, **p < 0.01; Mann–Whitney *U* test. **J** Flow cytometric analysis of Th1/Th17 cells in the dLNs of *Tcrb*^–/–^ mice (n = 3 mice per group) received CD4^+^ T cells (5 × 10^6^) from *Nedd4*^*f/f*^* and Rorc Cre*-*Nedd4*^f/f^ mice at day 8 after immunization. Data are representative of two to three independent experiments

To test whether Nedd4 regulates Th17-mediated autoimmunity we used EAE as a model in which the pathogenic Th17 response has been shown to play a key role in disease development [11, 33]. After tamoxifen-mediated deletion of Nedd4, we immunized *Nedd4*^*CreER*^ mice subcutaneously with an emulsion containing myelin oligodendrocyte glycoprotein peptide fragment 35–55 (MOG_35-55_) in complete Freund's adjuvant (CFA) to induce EAE. In contrast to *Nedd4*^*CreER*^ mice treated with vehicle corn oil or Rosa26-Cre-ERT2 mice treated with tamoxifen that developed severe disease, *Nedd4*^*CreER*^ mice treated with tamoxifen developed ameliorated EAE (Fig. 2A). To determine which cell type(s) is responsible for the phenotype, we used *Cd4 Cre-Nedd4*^*f/f*^, *Cd11c Cre-Nedd4*^f/f^, *LysM Cre-Nedd4*^f/f^, and *Cd19 Cre-Nedd4*^f/f^ mice. As shown in Fig. 2B–D, *Cd4 Cre-Nedd4*^f/f^, but not *Cd11c Cre-Nedd4*^f/f^, *LysM Cre-Nedd4*^f/f^ and *Cd19 Cre-Nedd4*^f/f^ mice develop EAE with significantly delayed onset and reduced severity. Our data strongly indicate that Nedd4 positively regulates the development of EAE in mice in a T cell-intrinsic manner.

It is believed that the EAE development is mediated by both Th1 and Th17 cells [34–36]. To analyze whether Nedd4 regulates Th1 or Th17 development during EAE, we first measure MOG_35-55_-specific T cell responses. To this end, we monitored ex vivo MOG_35–55_-specific T cell proliferation and cytokine production. Draining lymph node (dLN) cells from MOG_35–55_-immunized *Cd4 Cre-Nedd4*^*f/f*^ and *Cd4 Cre* mice (8 days after immunization) were labeled with CFSE, and stimulated with MOG_35–55_ for 5 days. T cell proliferation in response to MOG_35–55_ was significantly decreased in mice deficient for Nedd4 in T cells compared to those sufficient for Nedd4 (Supplementary Fig. 3A). These data are consistent with our previous report [20]. IL-17A, IL-6, GM-CSF, but not IFN-γ, in the culture supernatants were also significantly reduced in T cells lacking Nedd4 (Supplementary Fig. 3B). However, IL-1β and IL-21 were not detectable in this culture (Supplementary Fig. 3B). These data suggest that Nedd4 may be required for pathogenic Th17 responses. To confirm this data, we analyzed T cell responses in dLNs at the preclinical stage (day 8 after immunization). IFN-γ^+^ Th1 cells and IL-17^+^ Th17 cells were significantly increased in *Cd4 Cre* mice, whereas IL-17^+^ Th17 cells, but not Th1 cells, were defective in *Cd4 Cre-Nedd4*^*f/f*^ mice (Fig. 2E). In support of this finding, I-A^b^-MOG_35-55_ tetramer-positive IL-17^+^ cells were dramatically reduced in *Cd4 Cre-Nedd4*^*f/f*^ mice compared to those in *Cd4 Cre* controls (Fig. 2F). There was less lymphocyte infiltration and few CD4^+^IL-17^+^ T cells in the spinal cords of mice deficient for Nedd4 in T cells (Fig. 2G and H).

IL-17 is regulated by RORγt, and is mainly produced by type 3 innate lymphoid cells (ILC3) and T cells including Th17 cells [11, 37]. To further verify the importance of Nedd4 in the development of pathogenic Th17 cells and EAE, we crossed *Nedd4*^f/f^ mice with *Rorc Cre* mice, which express Cre recombinase under the control of the mouse RORγt promoter [38], to generate *Rorc Cre*-*Nedd4*^f/f^ mice. To avoid the confound effect of RORγt expression in DP thymocytes which impairs thymocyte development [39], we adoptively transferred CD4^+^ T cells from *Rorc Cre and Rorc Cre*-*Nedd4*^*f/f*^ mice to *Tcrb*^–/–^ recipients [11], and immunized the recipients with MOG_35-55_ in CFA. *Tcrb*^–/–^ mice receiving CD4^+^ T cells from *Rorc Cre*-*Nedd4*^*f/f*^ mice were found to be resistant to EAE induction, and displayed a defective Th17 response (Fig. 2I and J). To clarify whether the differentiation of Th1, Th2, and iTreg cells of *Rorc Cre*-*Nedd4*^*f/f*^ mice was affected by Nedd4 knocked out, we conducted in vitro differentiation experiments on naive CD4 + T cells isolated from *Rorc Cre and Rorc Cre*-*Nedd4*^*f/f*^ mice. In Supplementary Fig. 4, differentiation of pathogenic Th17, but not Th1, Th2, and iTreg cells, was significantly reduced in mice lacking Nedd4, thus providing further support of a crucial role for Nedd4 in pathogenic Th17 cell development in vivo.

### RORγt is a Nedd4-binding partner in T cells

**Fig. 3:**
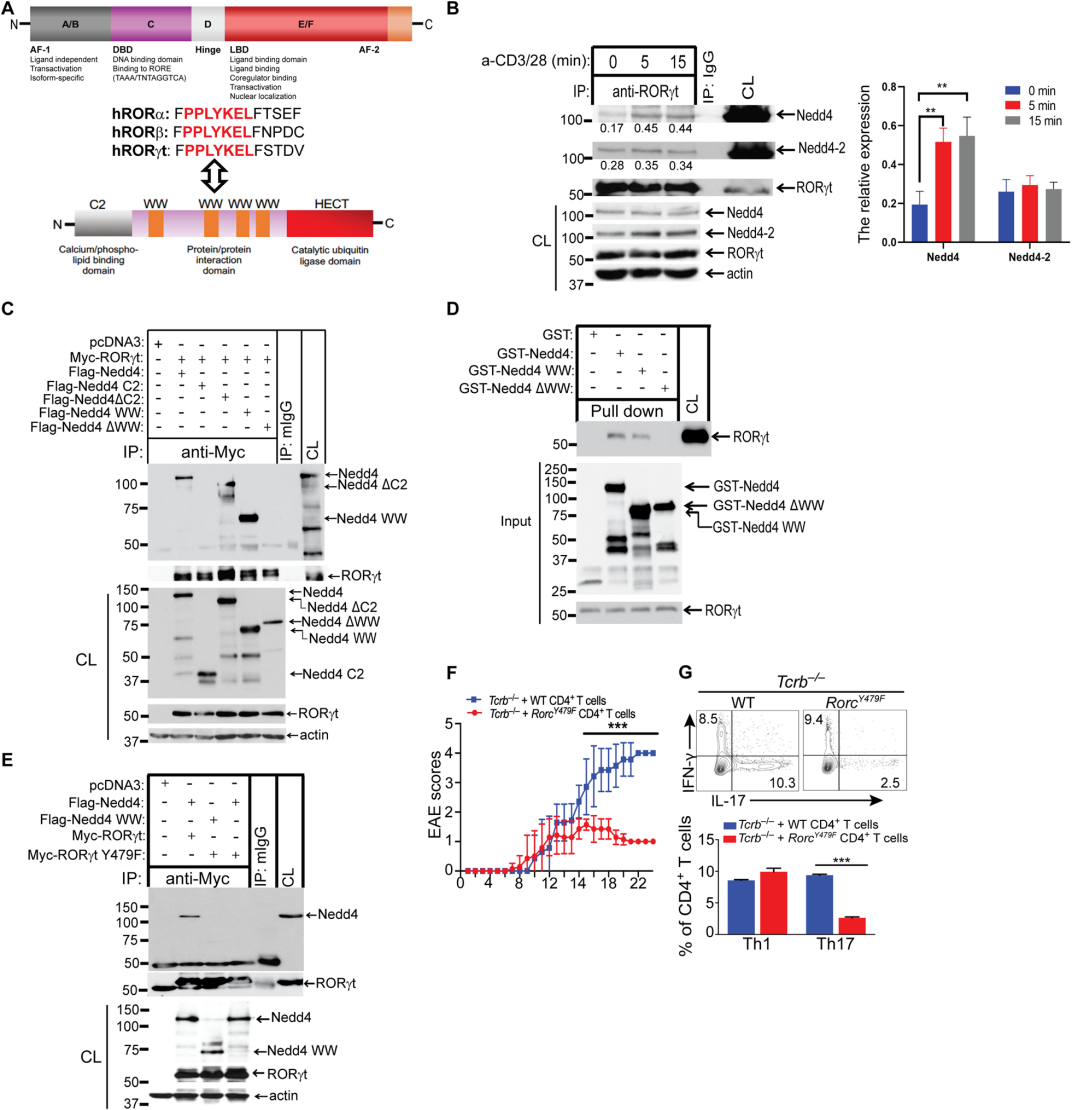
RORγt PPLY Motif Binds to the WW Domains of Nedd4. **A** Identification of a conserved Nedd4-binding motif in RORγt ligand-binding domain. Schematic diagram of the domain structure of RORs. Conserved sequence (PPLYKEL) of the AF2 domain among members of the ROR family (upper). Schematic diagram of the domain structure of Nedd4 (lower). **B** Immunoblot analysis of RORγt immunopreciptates of lysates of CD4^+^ T cells stimulated under Th17-biased condition (anti-CD3, anti-CD28, TGF-β, and IL-6) for three days, and restimulated with anti-CD3 and anti-CD28, with antibodies against Nedd4 and Nedd4-2. The relative expression of Nedd4 and Nedd4-2 to RORγt were shown under the WB bands. **p < 0.01; student *t* test. **C** Immunoblot analysis of Myc immunoprecipitates of HEK293T cells transfected with Flag-tagged Nedd4, Nedd4 WW domains, Nedd4 C2 domain, Nedd4∆C2, or Nedd4∆WW, together with Myc-tagged RORγt, with anti-Flag. **D** GST-Nedd4 and GST-Nedd4 WW domain pull-down assay using Th17 cell lysates. **E** Immunoblot analysis of Myc immunoprecipitates of lysates of HEK293T cells transfected with Flag-tagged Nedd4, and Myc-tagged RORγt or RORγt Y479F mutant, with anti-Flag. **F** EAE scores of *Tcrb*^–/–^ mice (n = 7 mice per group) receiving CD4^+^ T cells from WT and *Rorc*^*Y479F*^ mice immunized with immunization with MOG_35-55_ in CFA. ***p < 0.001; Mann-Whitney *U* test. **G** Flow cytometric analysis of Th1/Th17 cells in the dLNs of *Tcrb*^–/–^ mice (n = 3 mice per group) received CD4^+^ T cells (5 × 10^6^) from WT *and Rorc*^*Y479F*^ mice at day 8 after immunization. ***p < 0.001; student *t* test. Data are representative of three independent experiments for B-E, and two independent experiments for F and G

Our previous data have suggested the positive correlation between Nedd4 and RORγt expression in CD4^+^T cells of MS patients (Fig. 1F) and the effect of Nedd4 knockout in RORγt expressing CD4^+^ T cells of EAE on Th17 cell response (Fig. 2I, J), which suggest that Nedd4 is closely related to RORγt in CD4^+^ T cells. To search for the potential target for Nedd4 in Th17 cells, we first performed sequence analyses to identify the conserved domains known to be important for nuclear receptors. A PPLYKEL motif was identified at the carboxyl terminus of the RORγt ligand-binding domain, and it is conserved in all members of the ROR family (Fig. 3A). Interestingly PPLY is a potential binding motif of Nedd4 WW domains [18, 19] and PPxYxxL has been shown to be an extended PPxL motif that binds to the Nedd4 WW domains [18]. To test whether RORγt binds to Nedd4 or Nedd4 family E3 ubiquitin ligases, we performed a co-immunoprecipitation assay to define whether Nedd4 interacts with RORγt, the master transcription factors for Th17 cell differentiation. We found that Nedd4 constitutively associated with RORγt in Th17 cells, and this association was increased upon CD3/CD28 stimulation (Fig. 3B). We also found that Nedd4-2 bound to RORγt (Fig. 3B). Since mouse and human Nedd4 shares approximately 50 to 60% sequence homology with mouse and human Nedd4-2, it is still possible that Nedd4 and Nedd4-2 may have a redundant role in Th17 responses. To test whether this is the case, we generated a conditional *Nedd4-2* floxed allele, and crossed *Nedd4-2*^*f*/f^ mice to *Cd4 Cre* mice to generate *Cd4 Cre-Nedd4-2*^*f/f*^ mice (Supplementary Fig. 5A). Interestingly, mice deficient or sufficient for Nedd4-2 in T cells displayed normal T cell development, but it seemed that Nedd4-2 deficiency resulted in a reduction in naïve CD4^+^ T cells which is associated with an increase in memory CD4^+^ T cells (Supplementary Fig. 5B-H). Mice deficient or sufficient for Nedd4-2 in T cells displayed a similar EAE susceptibility, and a comparable TH17 response (Supplementary Fig. 5I and J). These data indicate that Nedd4-2 does not potentiate a pathogenic Th17 response.

To confirm whether the Nedd4 WW domains bind to RORγt, we transfected HEK293T cells with Flag-tagged Nedd4, Nedd4 WW domains, Nedd4 C2 domain, Nedd4 without C2 (Nedd4∆C2), or Nedd4 without the WW domains (Nedd4∆WW), together with Myc-tagged RORγt, and lysed in 0.5% NP40 lysis buffer. The cell lysates were immunoprecipitated with anti-Myc and blotted with anti-Flag antibodies. We found that full-length Nedd4 and the Nedd4 WW domains, but not Nedd4 C2 or Nedd4∆WW, bound to RORγt (Fig. 3C). In further support of this finding, GST-Nedd4 and GST-Nedd4 WW could pull-down RORγt when they were incubated with Th17 cell lysates (Fig. 3D). To further confirm whether RORγt PPLY motif binds to Nedd4, we mutated RORγt PPLY to PPLF, and transfected HEK293T cells with Flag-tagged Nedd4 or Nedd4 WW and Myc-tagged RORγt or RORγt Y479F mutant. Mutation of RORγt PPLY to PPLF abrogated the Nedd4-RORγt interaction (Fig. 3E). Therefore, our data collectively indicate that the Nedd4 WW domains bind to the RORγt PPLY motif. To verify the importance of the RORγt PPLY motif, we generated mice expressing RORγt Y479F (*Rorc*^*Y479F*^) by CRISPR Cas9 technology (Supplementary Fig. 6A–C). Interestingly, *Rorc*^*Y479F*^ mice phenocopied the mice deficient for RORγt which show defective thymocyte development and lack lymph nodes, but the expression of RORγt in thymocytes of *Rorc*^*Y479F*^ mice was not affected (Supplementary Fig. 7). Indeed, *Tcrb*^–/–^ mice receiving CD4^+^ T cells from *Rorc*^*Y479F*^ mice resulted in only very mild disease, with a defective Th17 response (Fig. 3F and G). The above results strongly supported the notion that binding of RORγt PPLY motif is essential for RORγt function.

### Nedd4 targets RORγt for K27-linked polyubiquitination upon TCR/CD28 stimulation which is required for its activation

**Fig. 4:**
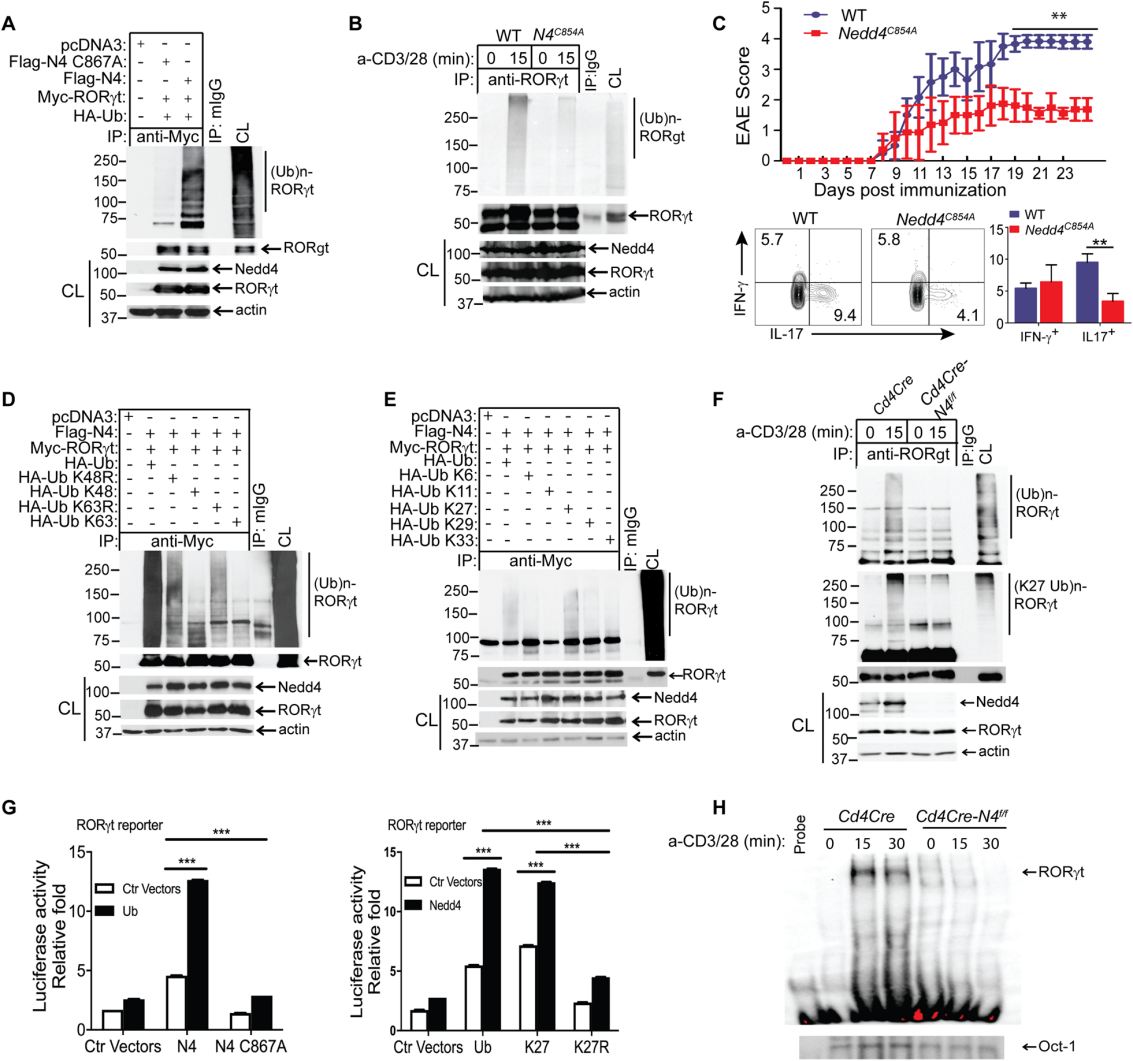
Nedd4 Targets RORγt for K27-Linked Polyubiquitination, thus Augmenting Its activity. **A** Anti-HA immunoblot analysis of Myc immunoprecipitates of lysates of HEK293T cells transfected with Flag-tagged Nedd4 or Nedd4 C867A mutant, together with Myc-tagged RORγt and HA-tagged ubiquitin. **B** Anti-ubiquitin immunoblot analysis of RORγt immunoprecipitates of lysates of CD4^+^ T cells from WT and *Nedd4*^*C854A*^ mice stimulated under Th17-biased condition for three days, and restimulated with anti-CD3 and anti-CD28 for 15 min or left unstimulated. **C** EAE scores and Th1/Th17 responses of WT and *Nedd4*^*C854A*^ mice (n = 3 mice per group) immunized with MOG_35-55_ in CFA. **p < 0.01; Mann–Whitney *U* test. **D** Anti-HA immunoblot analysis of Myc immunoprecipitates of lysates of HEK293T cells transfected with Flag-tagged Nedd4, Myc-tagged RORγt, and HA-tagged K48, K48R, K63, or K63R ubiquitin mutants. **E** Anti-HA immunoblot analysis of Myc immunoprecipitates of lysates of HEK293T cells transfected with Flag-tagged Nedd4, Myc-tagged RORγt, and HA-tagged K6, K11, K27, K29, or K33 ubiquitin mutants. **F** Anti-ubiquitin and anti-K27 ubiquitin immunoblot analyses of RORγt immunoprecipitates of Th17 cells from *Cd4 Cre* and *Cd4 Cre-Nedd4*^*f/f*^ mice restimulated with anti-CD3 and anti-CD28 for 15 min or left unstimulated. **G** RORγt reporter assay using HEK293T cells transfected with RORγt reporter gene, Flag-tagged Nedd4 or Nedd4 C867A together with HA-tagged ubiquitin (left panel), or alternatively, transfected with RORγt reporter gene, Flag-tagged Nedd4, HA-tagged K27, or K27R ubiquitin mutant (right panel). **H** ROR binding to the RORE at CNS2 using nuclear lysates of CD4^+^ T cells stimulated under Th17-biased condition for three days, and restimulated with anti-CD3 and anti-CD28, assessed by EMSA. Data are representative of three independent experiments for **A**–**F**, and two independent experiments for **G**

Next, we aimed to determine whether RORγt undergoes ubiquitination, and whether Nedd4 is the potential E3 ubiquitin ligase for RORγt. We transfected HEK293T cells with Flag-tagged Nedd4 or a Nedd4 C867A mutant which lacks E3 ubiquitin ligase activity [40], together with Myc-tagged RORγt and HA-tagged ubiquitin. Indeed, Nedd4, but not Nedd4 C867A, induced RORγt ubiquitination (Fig. 4A). To firmly validate these data, we generated knockin mice expressing Nedd4 C854A (equivalent to human Nedd4 C867A) by the CRISPR-Cas9 technology (Supplementary Fig. 6D–F). There was a similar level in the state of thymocytes and splenic T cells between WT and *Nedd4*^*C854A*^ mice (Supplementary Fig. 8). T cells expressing Nedd4 C854A abrogated RORγt ubiquitination (Fig. 4B). In keeping with this data, EAE severity was significantly reduced (Fig. 4C, upper panel), with an impaired Th17 response in mice expressing Nedd4 C854A (Fig. 4C, lower panel). Therefore, our data collectively indicate that Nedd4 is the E3 ubiquitin ligase for RORγt. Our data also indicate that the embryonic lethality observed in the conventional *Nedd4*^–/–^ mice may be independent of Nedd4 E3 ubiquitin ligase activity.

To define whether RORγt ubiquitination is K48- or K63-linked, we transfected HEK293T cells with Flag-tagged Nedd4, Myc-tagged RORγt, and HA-tagged K48, K48R, K63, or K63R ubiquitin. Surprisingly, RORγt did not undergo either K63- or K48-linked poly-ubiquitination (Fig. 4D). To further define which type of ubiquitin chain mediated by Nedd4 attaches to RORγt, we transiently transfected HEK293T cells with Flag-tagged Nedd4, Myc-tagged RORγt, and HA-tagged ubiquitin or ubiquitin mutants (K6, K11, K27, K29, and K33). Nedd4-mediated RORγt ubiquitination was only observed when WT ubiquitin or K27 ubiquitin was used (Fig. 4E), suggesting that RORγt undergoes K27-linked polyubiquitination. To confirm these data, we isolated naïve CD4^+^ cells from *Cd4 Cre* and *Cd4 Cre-Nedd4*^*f/f*^ mice, cultured them under pathogenic Th17-biased conditions for three days, and then restimulated them with anti-CD3 and anti-CD28. RORγt underwent polyubiquitination in T cells from *Cd4 Cre* but not *Cd4 Cre-Nedd4*^*f/f*^ mice (Fig. 4F, upper panel). Reprobing the same membrane with anti-K27 ubiquitin antibody showed that RORγt underwent K27-linked polyubiquitination in Th17 cells, and this K27-linked polyubiquitination was abrogated in Th17 cells lacking Nedd4 (Fig. 4F, lower panel).

Our data suggest that K27-linked polyubiquitination of RORγt mediated by Nedd4 increases its activity which results an increase in Th17-biased differentiation. In support of this notion, we first examined the expression of RORγt and IL-17 in naïve CD4^+^ T cells under Th17 differentiation conditions between mice deficient or sufficient for Nedd4. In Supplementary Fig. 9A, RORγt levels increased over time in both the *Cd4 Cre* and *Cd4 Cre-Nedd4*^*f/f*^ mice. However, there was no statistically significant difference in RORγt levels between the two groups. At the same time on day 5 of culture, the IL-17^+^ cells of RORγt^+^ CD4^+^ T cells in the Nedd4 knockout group was significantly lower than that in the control group (Supplementary Fig. 9B). This outcome indicates that the regulation of Th17 cells by Nedd4 is not achieved by altering the expression level of RORγt but by modulating the function of RORγt. Furthermore, co-transfecting HEK293T cells with Flag-tagged Nedd4, but not Nedd4 C867A, together with HA-tagged ubiquitin significantly augmented the activity of an RORγt reporter gene (Fig. 4G, left panel). In keeping with this, RORγt reporter activity was significantly increased when co-transfecting HEK293T cells with Flag-tagged Nedd4 together with K27 ubiquitin but not K27R ubiquitin (Fig. 4G, right panel). Both RORγt and RORα are highly homologous in their DNA-binding motif, the CNS2 site of *il17a* and *Il17f* genes contain two ROR response elements (RORE) that are conserved in human [33]. To further assess whether the potential regulation of CNS2 by RORs, performed an electrophoresis mobility shift assay (EMSA) with a CNS2 nucleotide probe containing a RORE [33]. Nuclear factors from CD4^+^ T cells expressing Nedd4 bound to this CNS probe, but this binding was impaired in the absence of Nedd4 (Fig. 4H). Our data indicate that K27-linked polyubiquitination of RORγt by Nedd4 enhances RORγt activation.

### Lysine 112 is the ubiquitination site of RORγt mediated by Nedd4

**Fig. 5:**
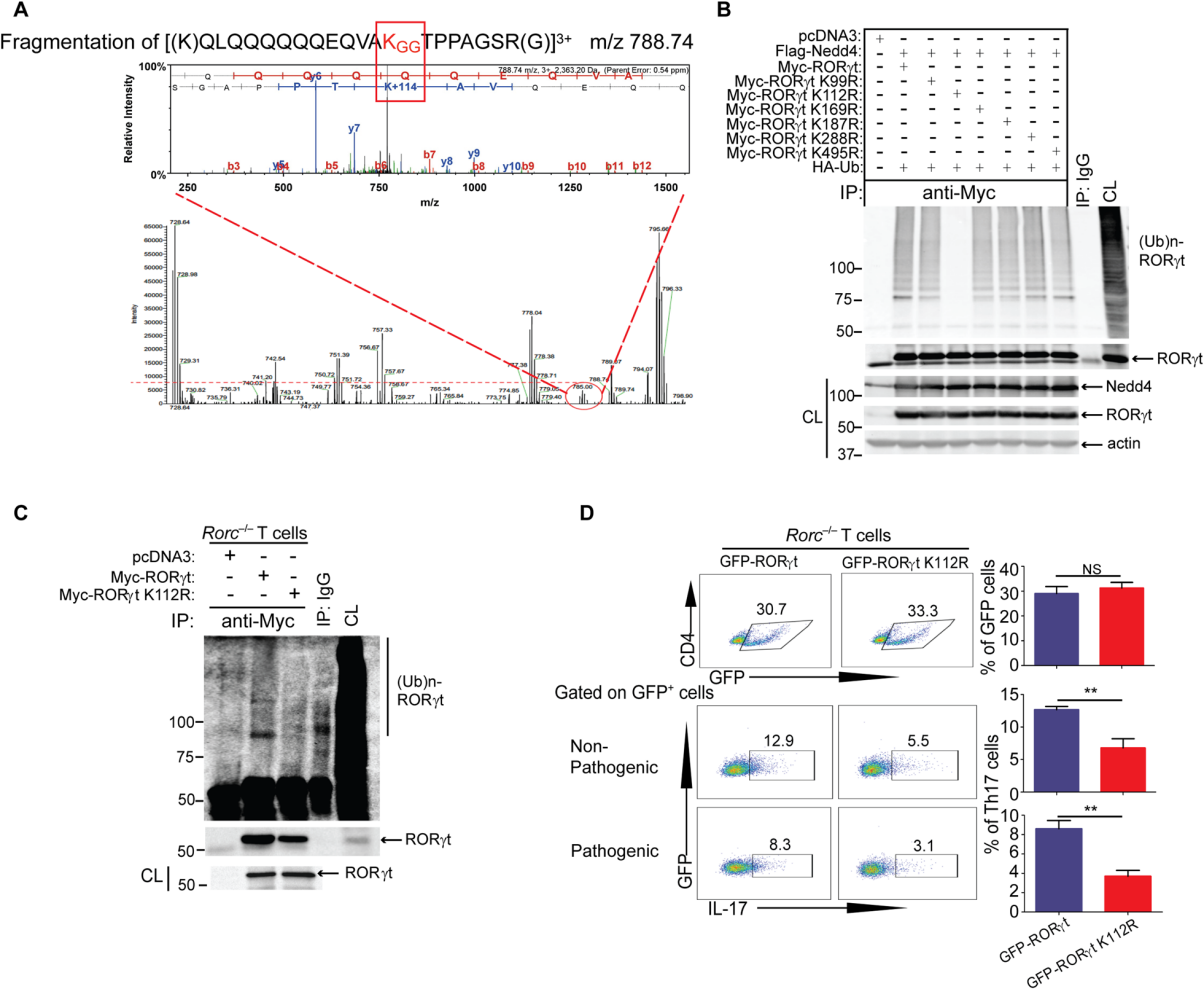
Lysine 112 Is the Ubiquitination Site in T Cells Mediated by Nedd4. **A** Mass spectrometric analysis of RORγt ubiquitination site(s) using purified Myc-tagged proteins from HEK293T cells transfected with Flag-tagged Nedd4, Myc-tagged RORγt, and HA-tagged ubiquitin. **B** Anti-HA immunoblot analysis of Myc immunoprecipitates of HEK293T cells transfected with Flag-tagged Nedd4, Myc-tagged RORγt or RORγt KXR (K99R, K112R, K169R, K288R and K495R), and HA-tagged ubiquitin. **C** Anti-ubiquitin immunoblot analysis of RORγt immunoprecipitates of CD4^+^ T cells from *Rorc*^–/–^ mice reconstituted with GFP-tagged RORγt or RORγt K112R, cultured under Th17-biased condition for two days, and restimulated with anti-CD3 and anti-CD28 for 15 min. **D** Th17 cell differentiation assay using naïve CD4^+^ T cells from *Rorc*^–/–^ mice (n = 3 mice per group) reconstituted with GFP-tagged RORγt or RORγt K112R. **p < 0.01; student *t* test. Data are representative of three independent experiments for B, and two independent experiments for **C** and **D**

To identify the potential ubiquitination site(s) of RORγt mediated by Nedd4, we transfected HEK293T cells with Flag-tagged Nedd4, Myc-tagged RORγt, and HA-tagged ubiquitin. Myc-tagged RORγt was purified using a Myc tagged protein purification kit (Clontech). The purified Myc-tagged RORγt was separated in an SDS-PAGE gel and the gel stained with Coomassie Blue. The bands corresponding to RORγt and above were excised, in gel digested by trypsin, and analyzed by conjunction with liquid chromatography (LC) tandem mass spectrometry on the orbitrap. The data were searched against human SwissProt database on MASCOT to identify the proteins and ubiquitination sites. Lysine 112 within RORγt was identified to attach to the ubiquitin chain (Fig. 5A). To further confirm that K112 is the RORγt ubiquitination site we generated an RORγt K112R mutant by site-directed mutagenesis. We transfected HEK293T cells with Flag-tagged Nedd4, Myc-tagged RORγt or RORγt K112R, and HA-tagged ubiquitin. The mutation at RORγt K112 completely abrogated RORγt ubiquitination induced by Nedd4 (Fig. 5B). For additional confirmation of this data, we reconstituted *Rorc*^–/–^ CD4^+^ T cells with a lentiviral vector encoding GFP tagged RORγt or RORγt K112R by lentiviral infection. *Rorc*^–/–^ CD4^+^ T cells reconstituted with GFP tagged RORγt or RORγt K112R were stimulated with anti-CD3 and anti-CD28, and lysed. The cell lysates were immunoprecipitated with anti- RORγt and blotted with anti-ubiquitin. Reconstituting *Rorc*^–/–^ CD4^+^ T cells with GFP tagged RORγt, but not RORγt K112R, restored RORγt ubiquitination (Fig. 5C), which correlated with enhanced Th17 cell differentiation (Fig. 5D). Taken together, our data indicate that Nedd4 ubiquitinates RORγt at K112 in Th17 cells upon TCR/CD28 stimulation which augments its activity.

## Discussion

Nedd4 and Itch are E3 ubiquitin ligases that ubiquitinate similar targets in vitro and thus are thought to function similarly [41]. Previous studies suggested that the role of Nedd4 in T cells may be related to T cell activation mediated by Cbl-b in Nedd4^–/–^ fetal liver chimeras [20, 21]. However, the role of Nedd4 in T cell responses has been hindered by the embryonic lethality of conventional Nedd4 knockout mice. To overcome this caveat, we have generated conditional Nedd4 knockout mouse strains, with specific deletion of Nedd4 in T cells, myeloid cells, DCs, and B cells. In this study, we report that loss of Nedd4 in T cells specifically impairs pathogenic and non-pathogenic Th17 responses, and Th17-mediated EAE development, while there is no obvious direct effect on the activation of CD4^+^ T cells. At the molecular level, Nedd4 binds to the PPLY motif within the ligand binding domain of RORγt, and targets RORγt at K112 for K27-linked polyubiquitination, which enhances RORγt activity. Therefore, inhibition of NEDD4 may represent a novel therapeutic approach for human MS.

ROR family members include RORα, RORβ, and RORγt and contain a PPLYKEL motif within the ligand-binding domain. It is well documented that the PPXY motif interacts with the WW domains of Nedd4 family members [18]. Through transfection and GST-Nedd4 pull-down assays, we confirm that the PPLY motif indeed binds to the Nedd4 WW domains. This is because: 1) the GST-Nedd4 WW domains can pulldown RORγt in Th17 cells, and the Nedd4 WW domains interact with RORγt in HEK293T cells; 2) Nedd4 lacking WW domains does not bind to RORγt; and 3) Nedd4 does not binds to an RORγt Y479F mutant (Fig. 3). RORγt PPLY motif have a critical role in the RORγt function and in the interaction between Nedd4 and RORγt, partly derived from *Rorc*^*Y479F*^ mice which have abnormal development of thymocytes and lack of lymph nodes (Supplementary Fig. 7) similar to *Rorc*^–/–^ mice. Meanwhile, we should also note that the results observed in *Rorc*^*Y479F*^ mice may be related to impairment of RORγt function, rather than interaction with Nedd4. This will be further clarified in our subsequent research on the exact binding site between Nedd4 and RORγt. Since mice deficient for Nedd4 or Nedd4-2 does not impair T cell development, our data argue that binding of RORγt to Nedd4 E3 ubiquitin ligases is crucial for the differentiation of Th17 cells. Importantly, *Tcrb*^–/–^ mice receiving CD4^+^ T cells from *Rorc*^*Y479F*^ mice are resistant to EAE induction and display a defective Th17 response (Fig. 3G and H). Furthermore, in the ex vivo differentiation of Th17 cells, the absence of Nedd4 did not alter RORγt expression, but still ultimately affected the expression of IL-17 in CD4^+^T cell (Fig. 1H and Supplementary Fig. 8). These data also strongly suggest the binding of Nedd4 E3 ubiquitin ligases are essential for RORγt function, rather than targeting RORγt for proteasome-mediated degradation. Since Nedd4 shares approximately 50–60% sequence homology with Nedd4-2, and both Nedd4 and Nedd4-2 bind RORγt, it is possible that other Nedd4 family E3 ubiquitin ligase(s) may also be involved in regulating RORγt. However, mice lacking Nedd4-2 in T cells do not impair Th17 responses and EAE development (Supplementary Fig. 5). These data indicate that although these two Nedd4 family members share 50–60% homology, they also have distinct substrates which mediate different functions [42]. In keeping with this notion, it was reported that both Itch and Nedd4-2 may regulate Th2 responses and Th2-mediated inflammatory disease via targeting JunB for ubiquitination [41, 43].

It is well documented that K48-linked polyubiquitination leads to proteasome-mediated degradation, whereas K63-linked polyubiquitination may alter the functions of the proteins possibly by conformation change [44]. It is less well defined how K27-linked polyubiquitination regulates biological functions. Several studies suggest that K27-linked polyubiquitination of proteins does not lead to degradation in the proteasome, but rather alters their functions [45–49]. RORγt can undergo K48- or K63-linked ubiquitination which leads to either proteasome-mediated degradation or altered function, both of which contribute to the development of Th17 cells [16, 50, 51]. Loss of deubiquitinase DUBA results in defective proteasome-mediated RORγt degradation, thus eliciting strong Th17 responses [49], whereas RORγt S92 and L93, which seem to mediate K63-linked polyubiquitination of RORγt at K69, is crucial for Th17 cell differentiation [51]. The E3 ubiquitin ligases that mediate RORγt ubiquitination is largely unknown, although TRAF-5 was reported to induce K63-linked polyubiquitination of RORγt [16]. Whether TRAF-5 is the E3 ubiquitin ligase for RORγt in vivo is unknown because this study did not use mice lacking TRAF-5 or expressing an inactive form of TRAF-5. Furthermore, whether RORγt undergoes other type(s) of ubiquitination with a different outcome(s) remains to be determined. Using several approaches including transfection of HEK193T cells with WT Nedd4 or Nedd4 C867A, together with RORγt and ubiquitin, and T cells from our newly-generated *Nedd4*^*C854A*^ mice, we collectively demonstrate that Nedd4 is the E3 ubiquitin ligase for RORγt which is crucial for Th17 cell differentiation. Indeed, mice lacking Nedd4 globally or in T cells, or expressing Nedd4 C854A, or lacking Nedd4 in Th17 cells, are resistant to EAE induction and show defective Th17 responses (Figs. 2 and 4C).

Our data has revealed that RORγt is ubiquitinated at K112 via a K27-polyubiquitin chain-dependent manner, and that this process is induced by Nedd4. K27-linked polyubiquitination of RORγt results in the up-regulation of RORγt activation, and this process requires Nedd4 E3 ubiquitin ligase activity. Therefore, we provide the first evidence that K27-linked polyubiquitination of RORγt increases its activity, thus enhancing both pathogenic and non-pathogenic Th17 responses, and the development of EAE. Our data also suggest that the embryonic lethality observed in conventional *Nedd4*^–/–^ mice is likely not due to its E3 ubiquitin ligase activity, but rather its adaptor functions mediated by Nedd4 WW and C2 domains.

Certainly, this study still had several limitations. Although we have clarified the domain of Nedd4 binding to RORγt and clarified the important role of the RORγt PPLY motif in its function, we have not yet explored the exact site of their binding, especially the molecular site of Nedd4 binding to the RORγt PPLY motif. To this end we explored Molecular Docking analysis and found multiple possible sites of Nedd4 binding to RORγt (Supplementary Fig. 10 and Supplementary Table 1). We will validate these sites one by one in future studies. In addition, we will determine how Nedd4 is activated in T cells during Th17 cell differentiation. The structural and functional analysis of Nedd4 indicates that under normal conditions, the activity of various Nedd4 E3 ligases is controlled through an autoinhibitory interaction of the N-terminal C2 domain with the C-terminal catalytic HECT domain [52, 53]. In this study, we found that the expression level of Nedd4 did not be changed after CD4^+^ T cell activation, which also indicated that the activation signal of CD4 + T cells mainly changed the active state of Nedd4 rather than its expression level. We will conduct an in-depth exploration of the specific mechanisms underlying Nedd4 activation during the differentiation of Th17 cells in future studies.

## Conclusion

Taken together, we have identified Nedd4 as a key E3 ubiquitin ligase that ubiquitinates the master transcription factor RORγt at K112 for Th17 cell development via attachment of the K27-linked polyubiquitin chains, which leads to the up-regulation of RORγt activation. Therefore, we have identified a novel mechanism by which the HECT E3 ubiquitin ligase Nedd4 regulates Th17 responses. Targeting NEDD4 in humans may offer a promising therapeutic strategy for the treatment of Th17-mediated autoimmune diseases, including multiple sclerosis.

## Supplementary Information

Additional file 1.

## Data Availability

The datasets used and/or analysed during the current study are available from the corresponding author on reasonable request.

## Abbreviations

MS: Multiple sclerosis
EAE: Experimental autoimmune encephalomyelitis
Nedd4: Neuronal precursor cell-expressed developmentally down-regulated 4
LBD: Ligand-binding domain
WT: Wild-type
PBMCs: Peripheral blood mononuclear cells
CHX: Cycloheximide
ES: Embryonic stem
ssODNs: Single strand oligo donor DNAs
LPLs: Lamina Propria lymphocytes
IELs: Intraepithelial lymphocytes
LC: Liquid chromatography
AGC: Automatic Gain Control
HCD: High energy collision-induced dissociation
RRMS: Relapsing–remitting multiple sclerosis
DN: Double negative
ILC3: Type 3 innate lymphoid cells
