# Correction: K27-linked RORγt ubiquitination by Nedd4 potentiates Th17-mediated autoimmunity

**DOI:** 10.1186/s12929-025-01136-8

**Published:** 2025-04-16

**Authors:** Qiuming Zeng, Hui Guo, Na Tang, Pranav S. Renavikar, Nitin J. Karandikar, Amy E. Lovett-Racke, Michael K. Racke, Chengkai Yan, Rong Tang, Sushmita Sinha, Krishnendu Ghosh, Jeremy P. Ryal, Song Ouyang, Min Chen, Foued Amari, Coppola Vincenzo, R. Marshall Pope, Yalan Li, Huan Yang, Wallace Y. Langdon, Jian Zhang

**Affiliations:** 1 Department of Neurology, Xiangya Hospital, Central South University, Changsha, Hunan 410008 People’s Republic of China; 2 Department of Pathology, The University of Iowa Roy J. and Lucille A. Carver College of Medicine, Iowa City, IA 52242 USA; 3 Department of Microbial Infection and Immunity, The Ohio State University, Columbus, OH 43210 USA; 4 Department of Neurology, The Ohio State University, Columbus, OH 43210 USA; 5 Genetically Engineered Mouse Modeling Core, The Ohio State University, Columbus, OH 43210 USA; 6 Department of Cancer Biology and Genetics, The Ohio State University, Columbus, OH 43210 USA; 7 Proteomics Facility, The University of Iowa Roy J. and Lucille A. Carver College of Medicine, Iowa City, Iowa, USA; 8 School of Biomedical Sciences, The University of Western Australia, Perth, Australia; 9 Clinical Research Center for Neuroimmune and Neuromuscular Disorders, Xiangya Hospital, Central South University, Changsha, Hunan 410008 People’s Republic of China; 10 National Clinical Research Center for Geriatric Disorders, Xiangya Hospital, Central South University, Changsha, Hunan 410008 People’s Republic of China

**Correction: Journal of Biomedical Science (2025) 32:26** 10.1186/s12929-025-01120-2

After publication of this article [1], the authors identified the affiliation of the first author is incorrect. The correct affiliations are shown in the author list and Author details section.

The original publication has been corrected.

## References

[1] Zeng Q, Guo H, Tang N, Renavikar PS, Karandikar NJ, Lovett-Racke AE, Racke MK, Yan C, Tang R, Sinha S, Ghosh K, Ryal JP, Ouyang S, Chen M, Amari F, Vincenzo C, Pope RM, Li Y, Yang H, Langdon WY, Zhang J. K27-linked RORγt ubiquitination by Nedd4 potentiates Th17-mediated autoimmunity. J Biomed Sci. 2025;32:26. 10.1186/s12929-025-01120-2. 39972304 10.1186/s12929-025-01120-2 PMC11841259

