# Supplementary material for: K27-linked RORγt ubiquitination by Nedd4 potentiates Th17-mediated autoimmunity
Source: J Biomed Sci. 2025 Feb 19;32:26. doi: 10.1186/s12929-025-01120-2 (PMC11841259; doi:10.1186/s12929-025-01120-2)
Supplement: Supplementary file 1 — Additional file 1. [file 12929_2025_1120_MOESM1_ESM.pdf]

Supplementary Table 1 Residues that can produce hydrogen bonding between NEDD4 and RORG

The residues of NEDD4	The residues of RORG
ASN-317	TYR-202
THR-388	ARG-206
PRO-395	ASP-213
SER-404	SER-214
GLN-415	TYR-216
ASN-427	GLN-221
MET-438	THR-234
ARG-451	SER-256
THR-454	ARG-286
ALA-497	HIS-449
GLN-790	ARG-457

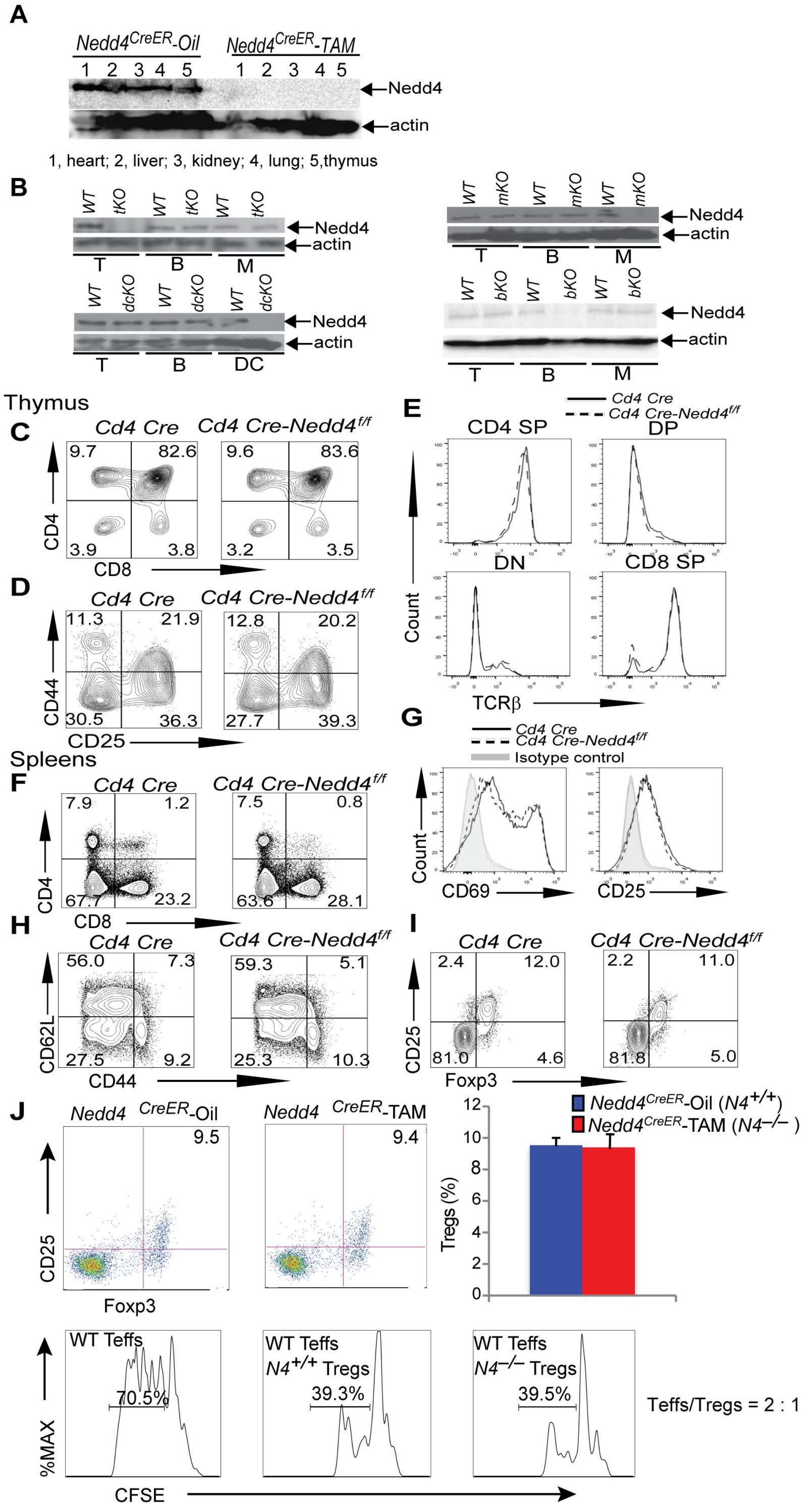
A
Nedd4CreER-Oil
Nedd4CreER-TAM
1 2 3 4 5
Nedd4
actin
1, heart; 2, liver; 3, kidney; 4, lung; 5,thymus
B
WT tKO
WT mKO
WT dcKO
WT bKO
T B M DC
Thymus
C
Cd4 Cre
Cd4 Cre-Nedd4f/f
9.7 82.6 3.9 3.8
9.6 83.6 3.2 3.5
CD4
CD8
D
11.3 21.9 30.5 36.3
12.8 20.2 27.7 39.3
CD44
CD25
E
CD4 SP
DP
DN
CD8 SP
Count
TCRβ
Spleens
F
7.9 1.2 67.7 23.2
7.5 0.8 63.6 28.1
G
Isotype control
CD69
CD25
H
56.0 7.3 27.5 9.2
59.3 5.1 25.3 10.3
CD62L
I
2.4 12.0 81.0 4.6
2.2 11.0 81.8 5.0
Foxp3
J
Nedd4CreER-Oil
Nedd4CreER-TAM
9.5
9.4
Nedd4CreER-Oil (N4+/+)
Nedd4CreER-TAM (N4−/−)
Tregs (%)
WT Teffs
70.5%
N4+/+ Tregs
39.3%
N4−/− Tregs
39.5%
%MAX
CFSE
Teffs/Tregs = 2 : 1

A

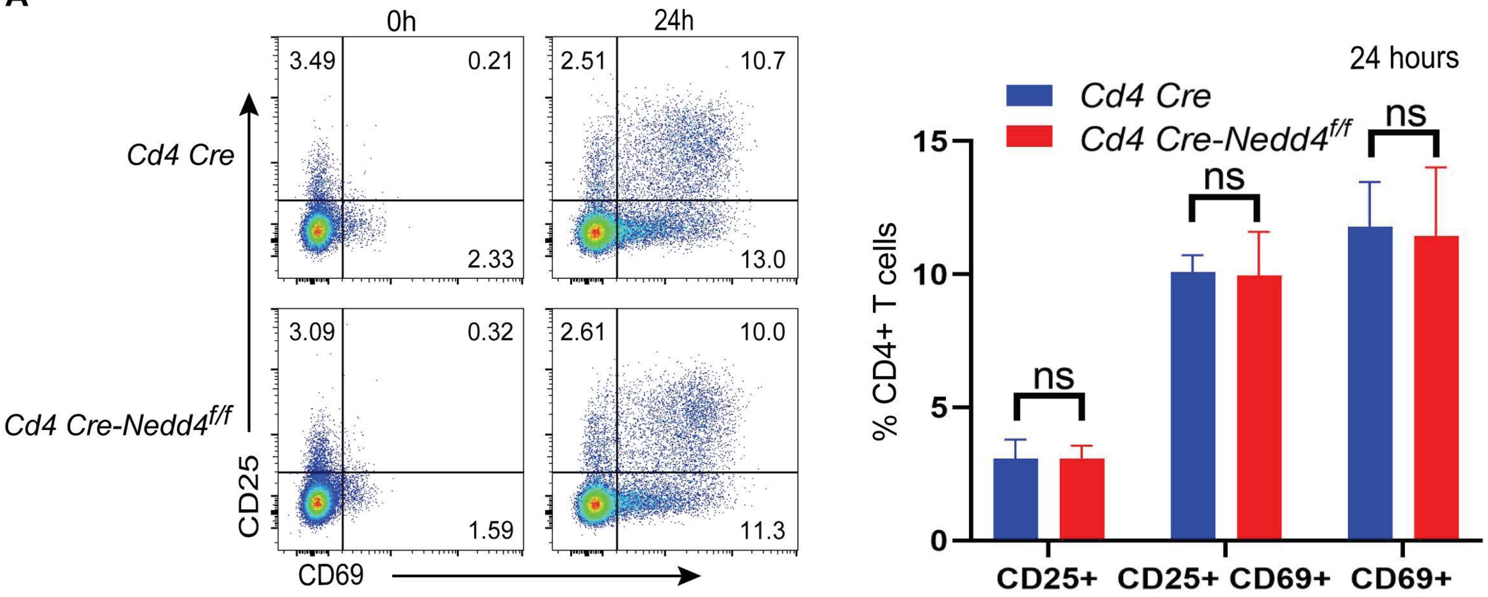
0h
24h
3.49
0.21
2.33
2.51
10.7
13.0
Cd4 Cre
3.09
0.32
1.59
2.61
10.0
11.3
Cd4 Cre-Nedd4f/f
CD25
CD69
24 hours
Cd4 Cre
Cd4 Cre-Nedd4f/f
ns
ns
ns
% CD4+ T cells
15
10
5
0
CD25+
CD25+ CD69+
CD69+

B

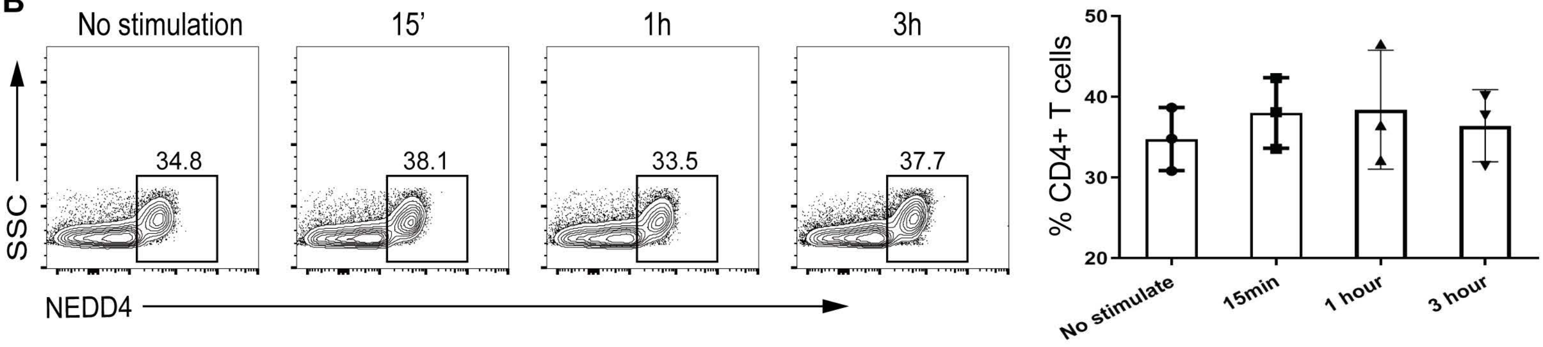
No stimulation
15'
1h
3h
34.8
38.1
33.5
37.7
SSC
NEDD4
% CD4+ T cells
50
40
30
20
No stimulate
15min
1 hour
3 hour

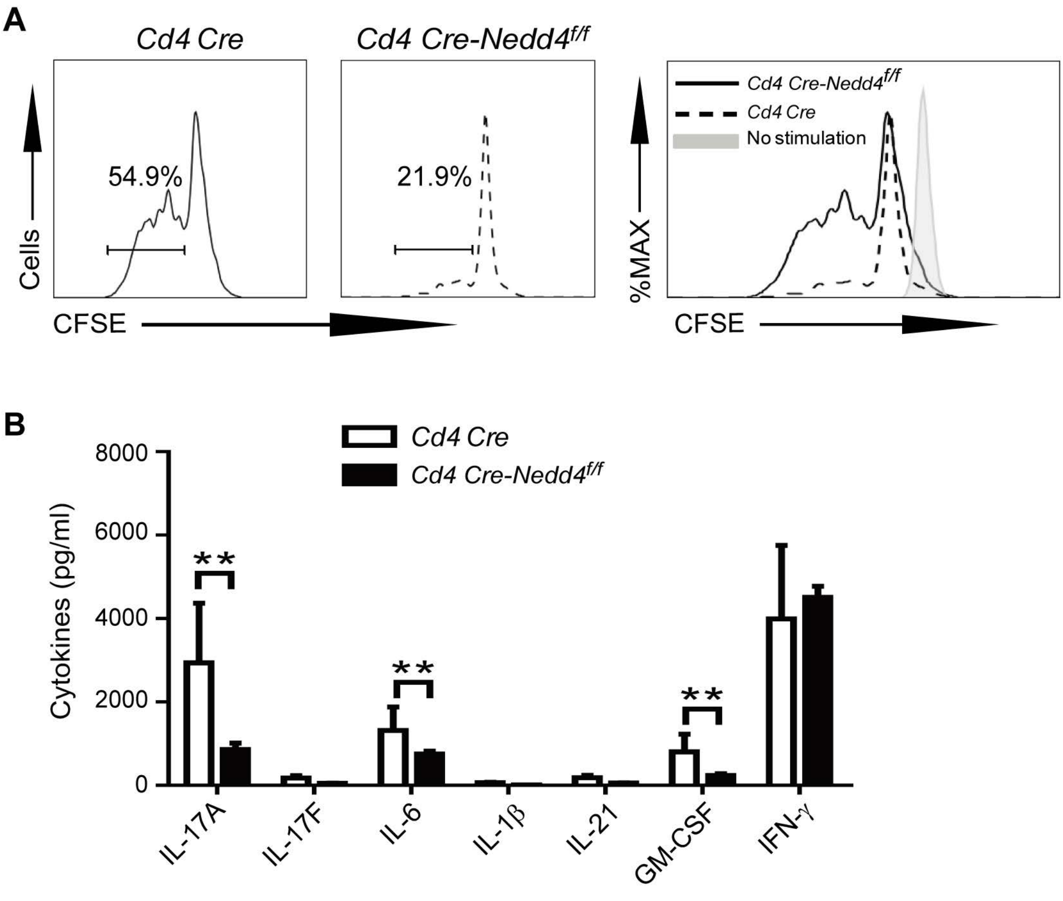
A
Cd4 Cre
Cd4 Cre-Nedd4f/f
54.9%
21.9%
Cells
CFSE
%MAX
Cd4 Cre-Nedd4f/f
Cd4 Cre
No stimulation
CFSE
B
Cd4 Cre
Cd4 Cre-Nedd4f/f
Cytokines (pg/ml)
8000
6000
4000
2000
0
**
**
**
IL-17A
IL-17F
IL-6
IL-1β
IL-21
GM-CSF
IFN-γ

Zeng, et al., Supplementary Fig. 4

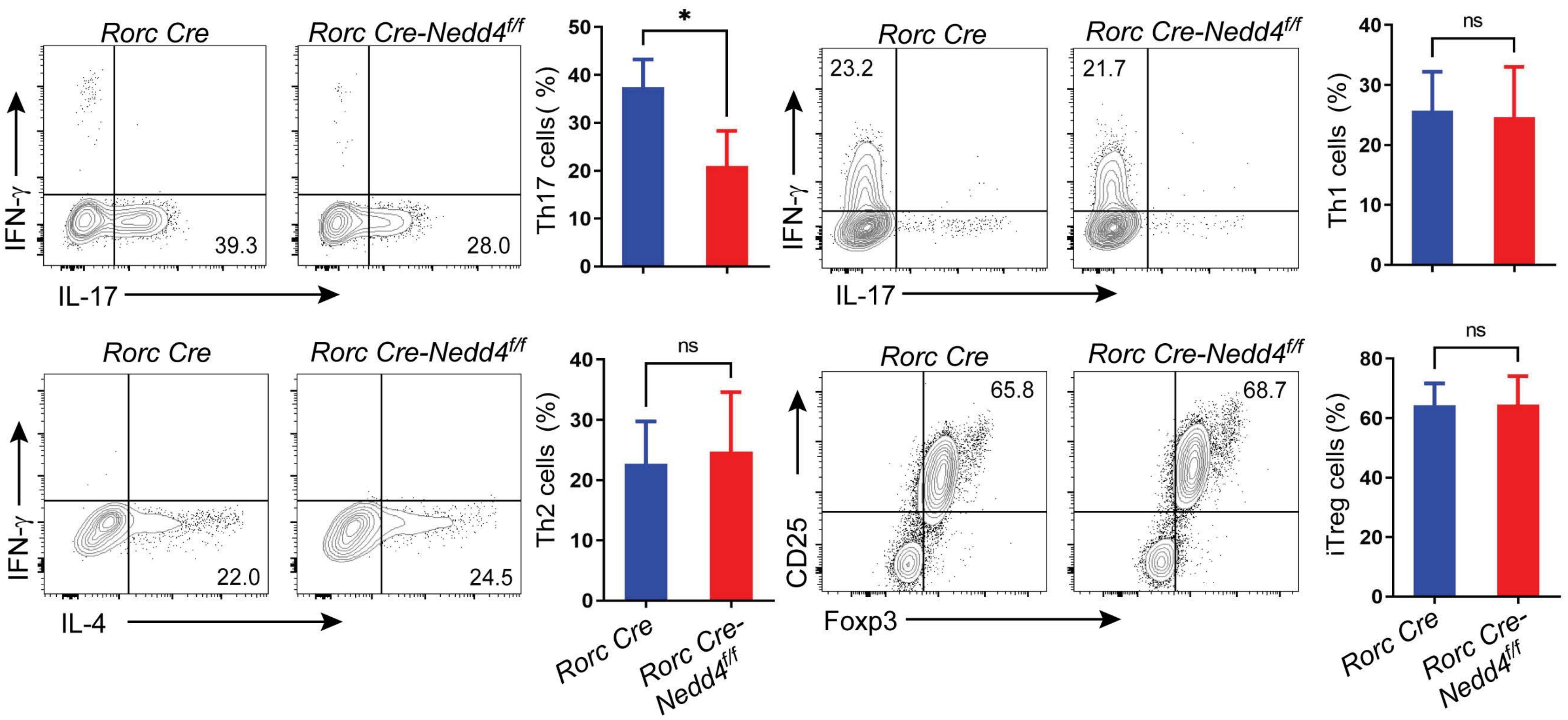

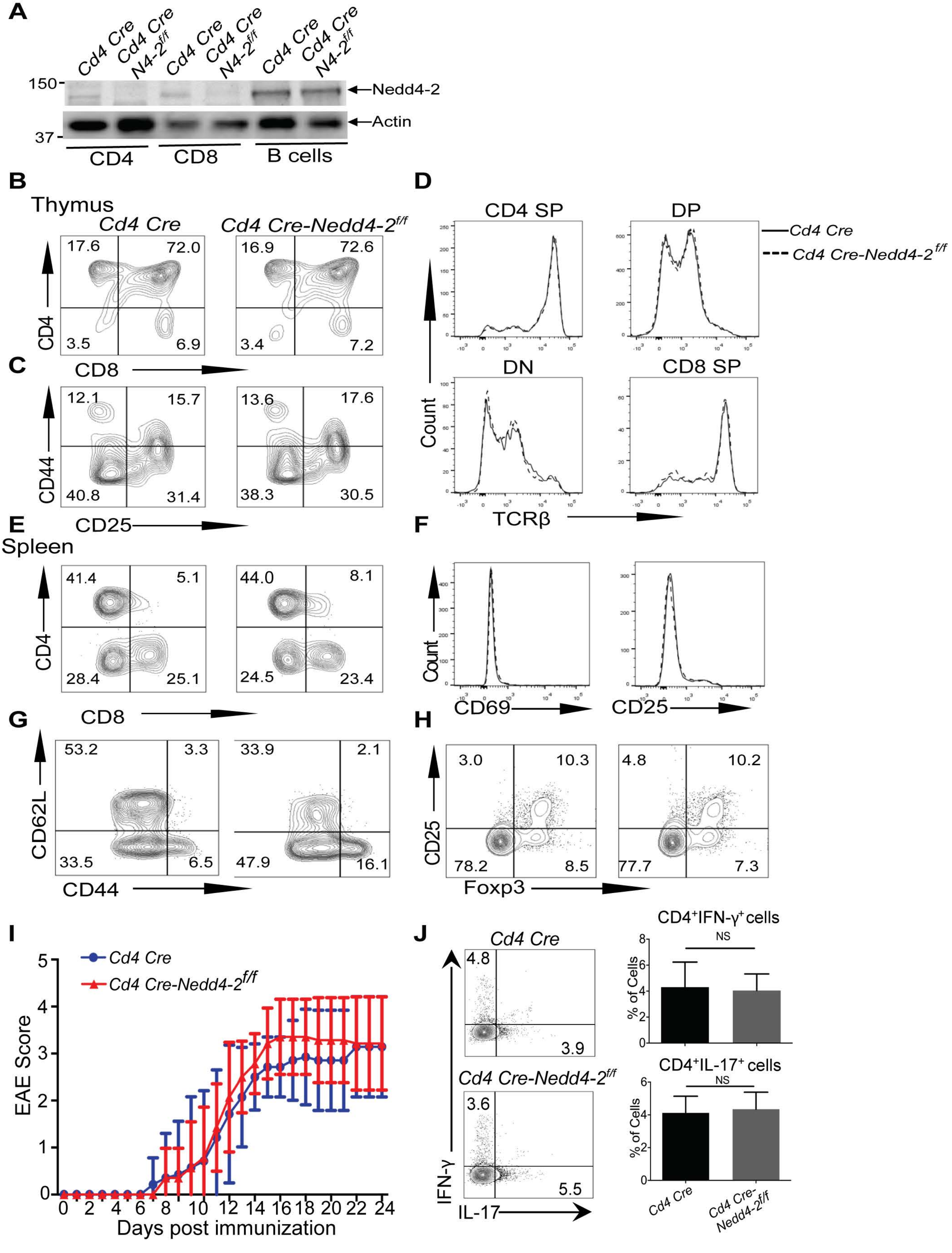
A
Cd4 Cre
Cd4 Cre N4-2f/f
B
Thymus
Cd4 Cre
Cd4 Cre-Nedd4-2f/f
C
D
CD4 SP
DP
DN
CD8 SP
TCRβ
Count
E
Spleen
F
CD69
CD25
G
CD62L
CD44
H
Foxp3
I
EAE Score
Days post immunization
J
CD4+IFN-γ+ cells
CD4+IL-17+ cells
NS
% of Cells
IFN-γ
IL-17

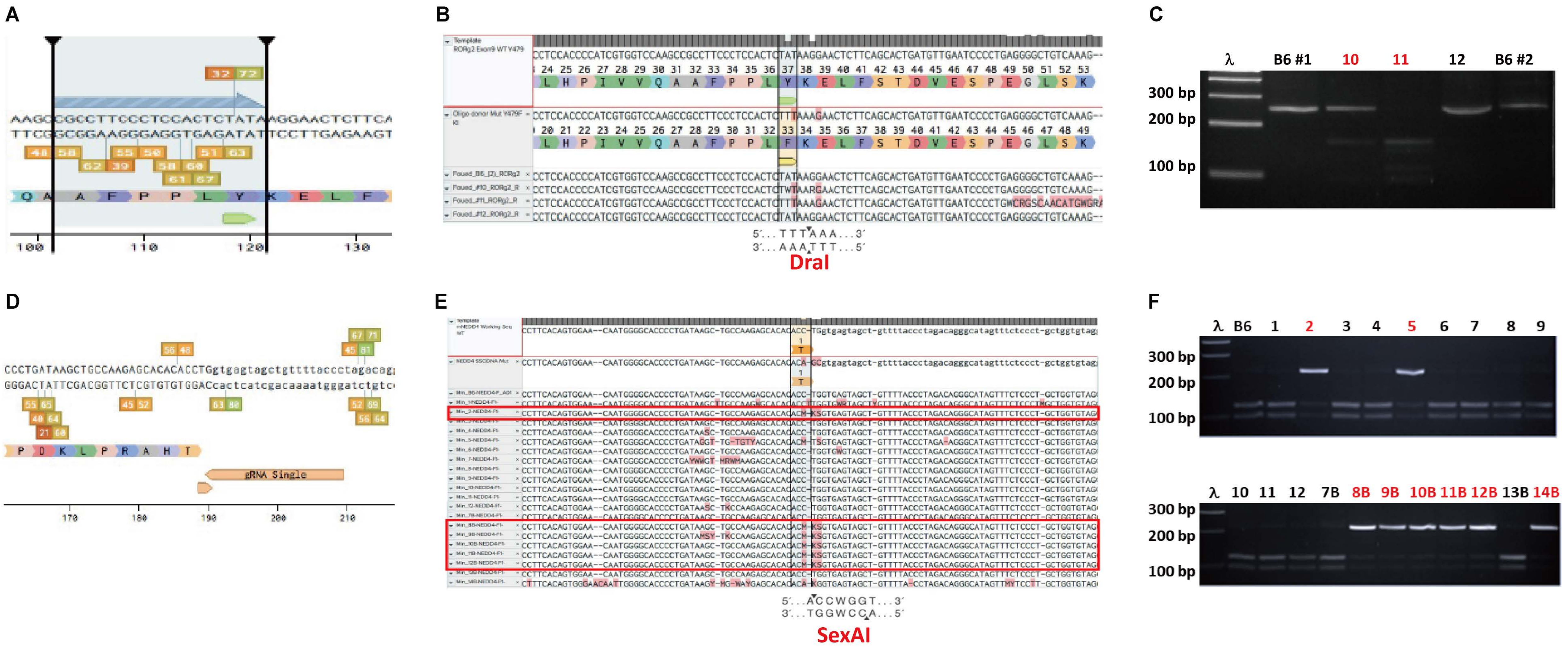
A
B
C
D
E
F
5′. . . T T T A A A . . . 3′
3′. . . A A A T T T . . . 5′
DraI
5′. . . A C C W G G T . . . 3′
3′. . . T G G W C C A . . . 5′
SexAI
λ
B6 #1
10
11
12
B6 #2
300 bp
200 bp
100 bp
B6
1
2
3
4
5
6
7
8
9
10
11
12
7B
8B
9B
10B
11B
12B
13B
14B
gRNA Single

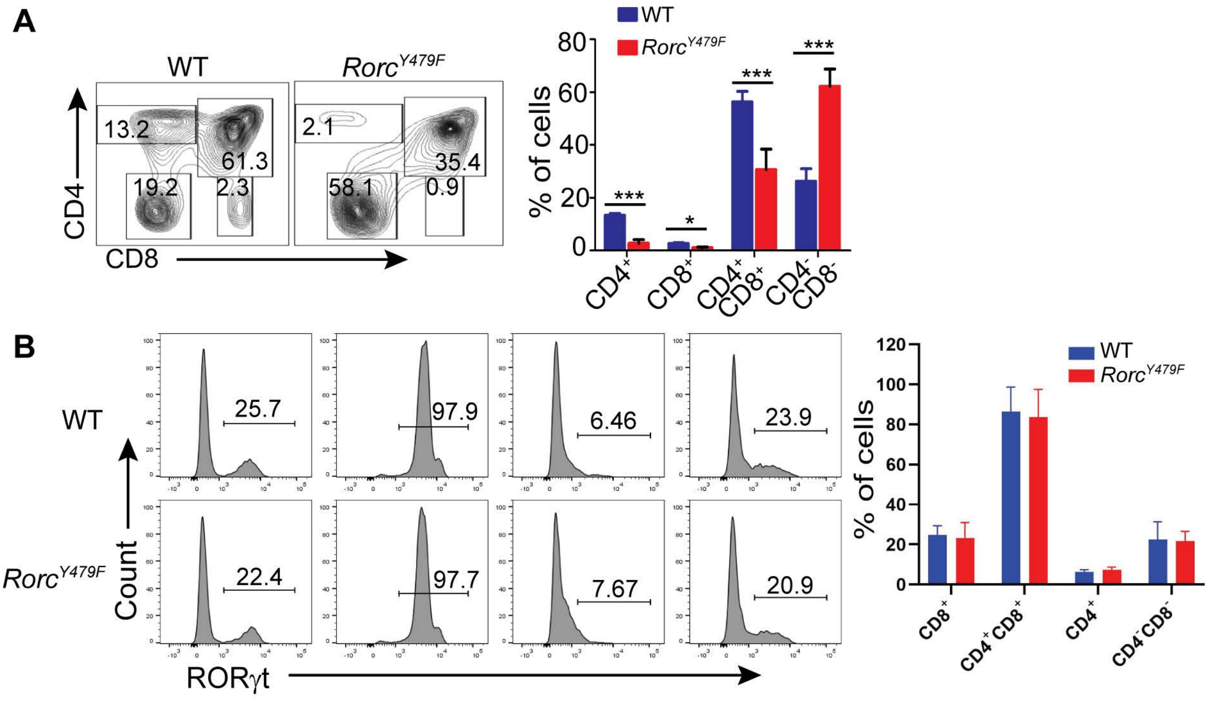
A
WT
$Rorc^{Y479F}$
CD4
CD8
13.2
61.3
19.2
2.3
2.1
35.4
58.1
0.9
WT
$Rorc^{Y479F}$
% of cells
80
60
40
20
0
***
*
***
***
CD4+
CD8+
CD4+ CD8+
CD4- CD8-
B
WT
$Rorc^{Y479F}$
Count
RORγt
25.7
97.9
6.46
23.9
22.4
97.7
7.67
20.9
% of cells
120
100
80
60
40
20
0
CD8+
CD4+CD8+
CD4+
CD4-CD8-

Zeng, et al., Supplementary Fig. 8

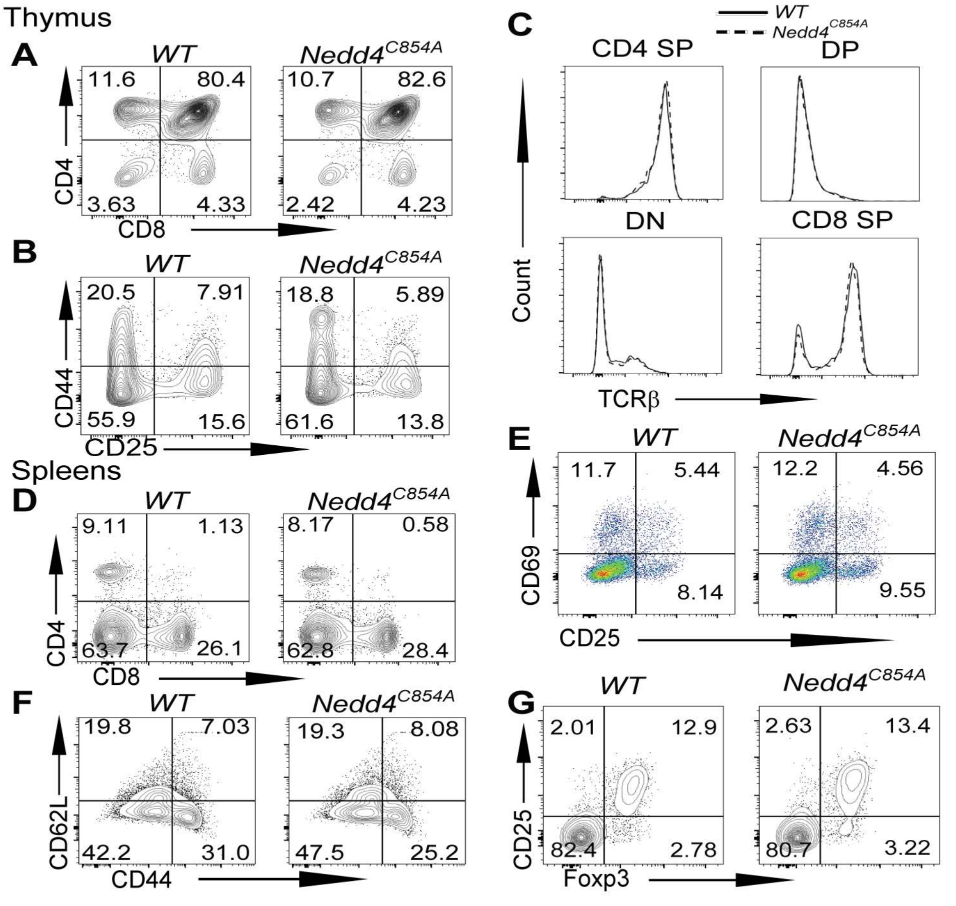

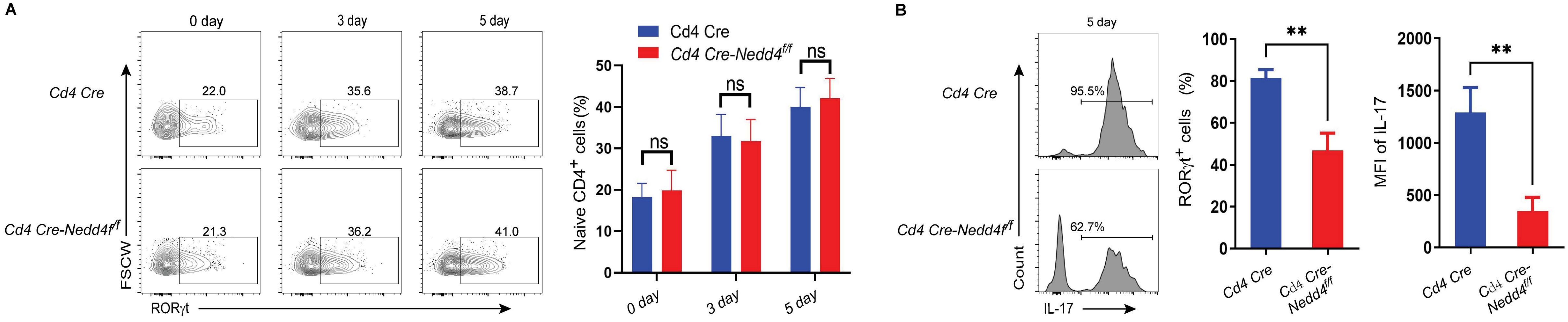
A
0 day
3 day
5 day
Cd4 Cre
Cd4 Cre-Nedd4f/f
22.0
35.6
38.7
21.3
36.2
41.0
FSCW
RORγt
Naive CD4+ cells (%)
ns
0 day
3 day
5 day
B
5 day
95.5%
62.7%
Count
IL-17
RORγt+ cells (%)
MFI of IL-17
**

Zeng, et al., Supplementary Fig. 10

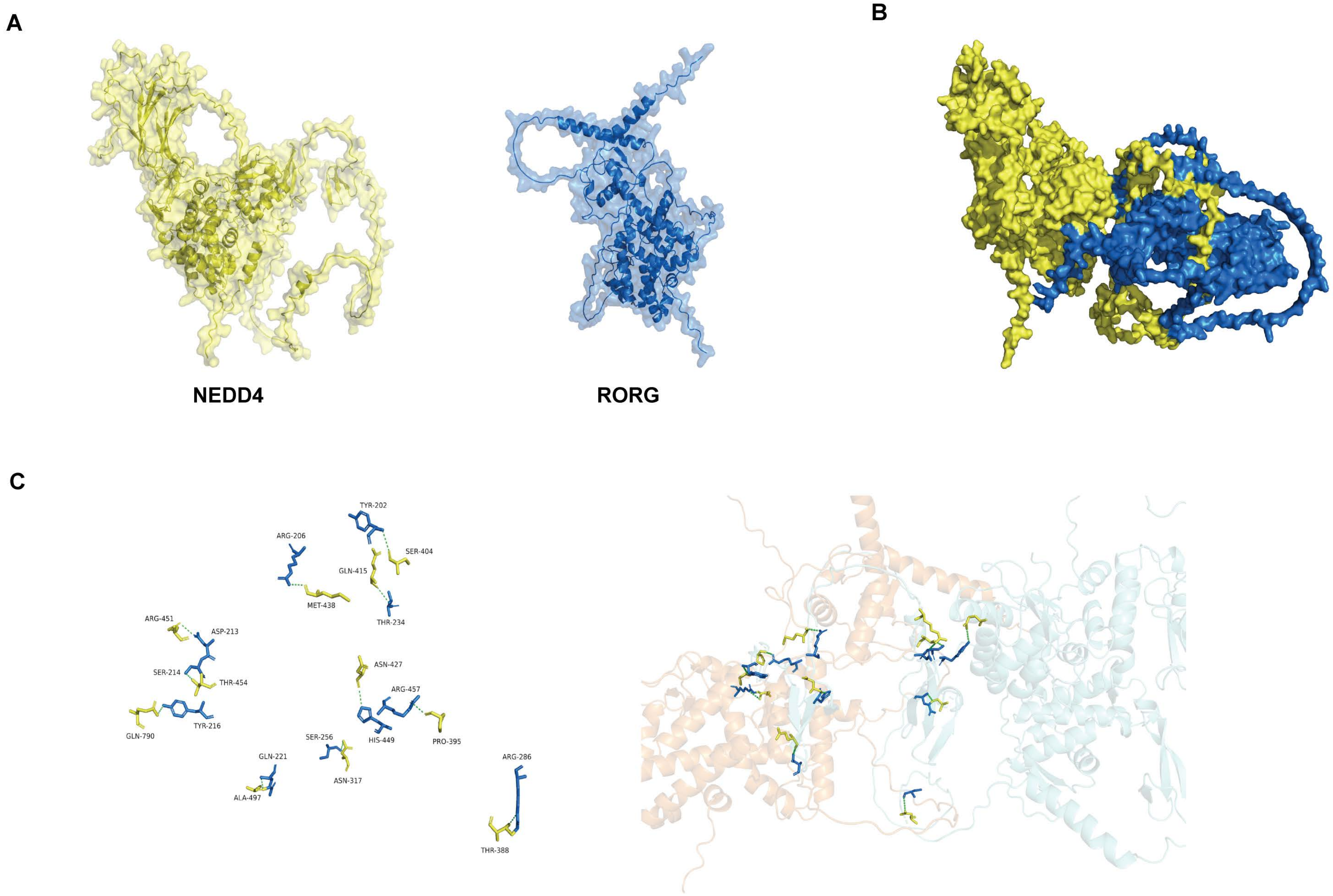

Supplementary Fig. 1. Generation of Nedd4 allele in mice and the state of thymocytes and splenic T cells from *Cd4 Cre* and *Cd4 Cre-Nedd4$^{f/f}$* mice

(A) Western blots of tissue homogenates from various organs from *Nedd4^{CreER}* mice pretreated with oil or tamoxifen.

(B) Western blots of immune cell subsets from *Cd4 Cre* (WT), *Cd4 Cre-Nedd4$^{f/f}$* (tKO), *LysM Cre* (WT), *LysM Cre-Nedd4$^{f/f}$* (mKO), *Cd11c Cre* (WT), *Cd11c Cre-Nedd4$^{f/f}$* (dcKO), *Cd19 Cre* (WT), and *Cd19 Cre-Nedd4$^{f/f}$* (bKO) mice. Peritoneal macrophages were stimulated with LPS (50 ng/ml) overnight. DC were generated from BM cells in the presence of GM-CSF, and purified using a DC isolation kit.

(C) Distributions of $CD4^+$ SP, $CD8^+$ SP, $CD4^+CD8^+$ DP, and $CD4^-CD8^-$ DN thymocyte populations of *Cd4 Cre* and *Cd4 Cre-Nedd4$^{f/f}$* mice (n = 3 per group).

(D) Distributions of DN1, DN2, DN3, and DN4 thymocyte populations of *Cd4 Cre* and *Cd4 Cre-Nedd4$^{f/f}$* mice (n = 3 per group).

(E) Expression of TCRβ in DN, DP, $CD4^+$ SP, and $CD8^+$ SP thymocytes of *Cd4 Cre* and *Cd4 Cre-Nedd4$^{f/f}$* mice (n = 3 per group).

(F) Distributions of splenic $CD4^+$ and $CD8^+$ T cells of *Cd4 Cre* and *Cd4 Cre-Nedd4$^{f/f}$* mice (n = 3 per group).

(G) Expression of activation marker CD69 and CD25 in splenic $CD4^+$ T cells of *Cd4 Cre* and *Cd4 Cre-Nedd4$^{f/f}$* mice (n = 3 per group).

(H) State of memory vs. naïve $CD4^+$ T cells in the spleens of *Cd4 Cre* and *Cd4 Cre-Nedd4$^{f/f}$* mice (n = 3 per group).

(I) Percentages of $CD4^{+}CD25^{+}Foxp3^{+}$ Tregs of *Cd4 Cre* and *Cd4 Cre-Nedd4$^{f/f}$* mice (n = 3 per group).

(J) Percentage and suppressive activity of Tregs in *Nedd4^{CreER}* mice pretreated with tamoxifen or corn oil (n = 3 mice per group).

Supplementary Fig.2 The activation of naïve $CD4^{+}$ T cell from *Cd4 Cre-Nedd4$^{f/f}$* mice and the Nedd4 expression of $CD4^{+}$ T cell after activation in WT mice.

(A) Naïve $CD4^{+}$ T cells from *Cd4 Cre and Cd4 Cre -Nedd4$^{f/f}$* mice were isolated by the Naïve $CD4^{+}$ T Cell Isolation Kit. Naïve $CD4^{+}$ T cells were stimulated with CD3 and CD28 for 24 hours (n = 3 mice per group). The CD25 and CD69 of Naïve $CD4^{+}$ T cells were detected by flow cytometry.

(B) Naïve $CD4^{+}$ T cells from WT mice were isolated by the Naïve $CD4^{+}$ T Cell Isolation Kit. Naïve $CD4^{+}$ T cells were stimulated with CD3 and CD28 for 15’, 1h and 3h (n = 3 mice per group).

Supplementary Fig. 3 Antigen-specific T cell proliferation and cytokine production of *Cd4 Cre* and *Cd4 Cre-Nedd4$^{f/f}$* mice

(A) dLN cells from *Cd4 Cre* and *Cd4 Cre-Nedd4f/f* mice (3 mice per group) were labeled with CFSE, and cultured in the presence of MOG_{35-55} (20 μg/ml) for 72 h, and surface-stained with anti-CD4. T cell proliferation was determined by flow cytometry.

(B) The supernatants collected from (A) was subjected for ELISA for cytokine production. ** $p < 0.01$, student t test.

Supplementary Fig. 4 The differentiation assays of naïve CD4+ T cells in *Rorc Cre and Rorc Cre-Nedd4$^{f/f}$* mice

Pathogenic Th17, Th1, Th2, and iTreg cell differentiation assays using naïve $CD4^+$ T cells from *Rorc Cre and Rorc Cre-Nedd4$^{f/f}$* mice (n = 3 mice per group). * $p < 0.05$, student *t* test.

Supplementary Fig. 5 The state of thymocytes and splenic T cells from *Cd4 Cre* and *Cd4 Cre-Nedd4-2$^{f/f}$* mice and EAE Induction

(A) Western blots of lysates of $CD4^+$, $CD8^+$ T cells, and B cells from *Cd4 Cre* and *Cd4 Cre-Nedd4-2$^{f/f}$* (*Cd4 Cre-N4-2$^{f/f}$*) mice (n = 3 per group).

(B) Distributions of $CD4^+$ SP, $CD8^+$ SP, $CD4^+CD8^+$ DP, and $CD4^-CD8^-$ DN thymocyte populations of *Cd4 Cre* and *Cd4 Cre-N4-2$^{f/f}$* mice (n = 3 per group).

(C) Distributions of DN1, DN2, DN3, and DN4 thymocyte populations of *Cd4 Cre* and *Cd4 Cre-N4-2$^{f/f}$* mice (n = 3 per group).

(D) Expression of TCRβ in DN, DP, $CD4^+$ SP, and $CD8^+$ SP thymocytes of *Cd4 Cre* and *Cd4 Cre-N4-2$^{f/f}$* mice (n = 3 per group).

(E) Distributions of splenic $CD4^+$ and $CD8^+$ T cells of *Cd4 Cre* and *Cd4 Cre-N4-2$^{f/f}$* mice (n = 3 per group).

(F) Expression of activation marker CD69 and CD25 in splenic CD4$^+$ T cells of *Cd4 Cre* and *Cd4 Cre-N4-2$^{f/f}$* mice (n = 3 per group).

(G) State of memory vs. naïve CD4$^+$ T cells in the spleens of *Cd4 Cre* and *Cd4 Cre-N4-2$^{f/f}$* mice (n = 3 per group).

(H) Percentages of CD4$^+$CD25$^+$Foxp3$^+$ Tregs of *Cd4 Cre* and *Cd4 Cre-N4-2$^{f/f}$* mice.

(I) EAE score of *Cd4 Cre* and *Cd4 Cre-Nedd4-2$^{f/f}$* mice (n = 7 mice per group).

(J) Flow cytometric analysis of CD4$^+$IFN-γ$^+$ Th1 and CD4$^+$IL-17$^+$ Th17 cells *Cd4 Cre* and *Cd4 Cre-Nedd4-2$^{f/f}$* mice (n = 3 per group) at day 8 after immunization.

Data are representative of three independent experiments for B-E, and two independent experiments for F-J.

Supplementary Fig. 6 Generation of *Rorc^{Y479F}* and *Nedd4^{C854A}* knockin mouse strains

CRISPR/Cas9 targeting and strategy to mutate Y479 (TAT) to F479 (TTT) of the Rorc mouse gene. Shown sequences are matched to the UCSC genome browser at https://genome.ucsc.edu on Mouse June 2020 (GRCm39/mm39) Assembly.

(A) The selected gRNA (Benchling on-target and off-target scores 32.2 and 72.2, respectively) drives Cas9 to cut in between residues 94,304,607 (T) and 94,304,608 (A) of the TAT triplet encoding for Y479.

(B) The designed 150bp ssODN that spans the 94,304,508 - 94,304,657 region of Chr3 (+ strand) (sequence in Methods Details) to introduce the mutation of TAT to TTT contains also the mutation of G 94,304,612 to A and introduces a DraI restriction site (TTTAAA), which can be used for genotyping purposes.

(C) 252 bp PCR products (spanning region 94,304,452 - 94,304,703) from 2 wild type C57/Bl6Tac controls (B6#1 and B6#2) and from 3 male potential founders were digested with DraI and sequenced. Correctly targeted mice show 158 bp and 94 bp fragments following DraI digestion. Mice devoid of additional undesired mutations such as #10 were used for colony propagation. CRISPR/Cas9 targeting and genotyping strategy to mutate C854 (CTG) to A854 (ACG) of the Nedd4 mouse gene. Shown sequences are matched to the UCSC genome browser at https://genome.ucsc.edu on Mouse June 2020 (GRCm39/mm39) Assembly.

(D) The selected gRNA (Benchling on-target and off-target scores 63.2 and 80.5, respectively) drives Cas9 to cut in between residues 72,653,763 (T) and 72,653,764 (G).

(E) The designed 150bp ssODN that spans the 72,653,689 - 72,653,838 region of Chr9 (+ strand) (sequence in Methods Details) to introduce the mutation of CTG to ACG contains also the mutation of C 72,653,759 to A and disrupts a SexAI restriction site (ACCWGGT), which can be used for genotyping purposes.

(F) 223 bp PCR products (spanning region 72,653,763 - 72,653,985) from 1 wild type C57/Bl6Tac controls (B6) and from 20 F1 heterozygous progeny from founder #3 were digested with SexAI and sequenced. Correctly targeted mice (in red) show a product of 223 bp due to the loss of the SexAI site. Mice devoid of additional undesired mutations were used for colony propagation.

Supplementary Fig. 7 Flow cytometric analysis of thymocyte compositions and RORγt expression of WT and *Rorc*^{Y479F} mice

(A) Flow cytometric analysis of thymocyte compositions (n = 3 mice per group). * $p < 0.05$, *** $p < 0.001$, student *t* test.

(B) The RORγt expression of thymocyte cells (n = 3 per group).

Supplementary Fig. 8 The state of thymocytes and splenic T cells from WT and *Nedd4*^{C854A} mice

(A) Distributions of $CD4^+$ SP, $CD8^+$ SP, $CD4^+CD8^+$ DP, and $CD4^-CD8^-$ DN thymocyte populations of WT and *Nedd4*^{C854A} mice (n = 3 per group).

(B) Distributions of DN1, DN2, DN3, and DN4 thymocyte populations of WT and *Nedd4*^{C854A} mice (n = 3 per group).

(C) Expression of TCRβ in DN, DP, $CD4^+$ SP, and $CD8^+$ SP thymocytes of WT and *Nedd4*^{C854A} mice (n = 3 per group).

(D) Distributions of splenic $CD4^+$ and $CD8^+$ T cells of WT and *Nedd4*^{C854A} mice (n = 3 per group).

(E) Expression of activation marker CD69 and CD25 in splenic $CD4^+$ T cells of WT and *Nedd4*^{C854A} mice (n = 3 per group).

(F) State of memory vs. naïve $CD4^+$ T cells in the spleens of WT and *Nedd4*^{C854A} mice (n = 3 per group).

(G) Percentages of $CD4^+CD25^+Foxp3^+$ Tregs of WT and *Nedd4*^{C854A} mice.

Supplementary Fig. 9 RORγt and IL-17 of naïve CD4+ T cells from *Cd4 Cre* and *Cd4 Cre-Nedd4*$^{f/f}$ mice in vitro Th17 differentiation

(A) Naïve $CD4^+$ T cells from *Cd4 Cre and Cd4 Cre -Nedd4*$^{f/f}$ mice were isolated by the Naïve $CD4^+$ T Cell Isolation Kit. Flow cytometric analysis of $RORγt^+$ CD4+ T cells of *Cd4 Cre* and *Cd4 Cre-Nedd4*$^{f/f}$ mice at 0d, 3d and 5d under Th17 differentiation condition. (n = 3 per group).

(B) The frequency of IL-17^+ cells in $RORγt^+$ CD4+ T cells of *Cd4 Cre* and *Cd4 Cre-Nedd4*$^{f/f}$ mice at 5 day under Th17 differentiation condition. (n = 3 per group). ** $p < 0.01$, student *t* test.

Supplementary Fig. 10 Molecular Docking analysis between NEDD4 and RORG

(A) Three-dimensional structure of the protein NEDD4 and RORG.

(B) Preferential binding mode between NEDD4 (yellow) and RORG (blue) according to a molecular docking simulation using GRAMM.

(C) Magnified view of the hydrogen bonds between NEDD4 and RORG

binding site amino acids demonstrated by Pymol (the residues of NEDD4 marked by yellow, the residues of RORG marked by blue and the hydrogen bonds are shown as green dashed lines).
